# CHEXVIS: a tool for molecular channel extraction and visualization

**DOI:** 10.1186/s12859-015-0545-9

**Published:** 2015-04-16

**Authors:** Talha Bin Masood, Sankaran Sandhya, Nagasuma Chandra, Vijay Natarajan

**Affiliations:** 1 Department of Computer Science and Automation, Indian Institute of Science, Bangalore, 560012 India; 2 Department of Biochemistry, Indian Institute of Science, Bangalore, 560012 India; 3 Supercomputer Education and Research Centre, Indian Institute of Science, Bangalore, 560012 India

**Keywords:** Biomolecular channels, Ion channels, Pores, Transmembrane pores, Alpha complex, Visualization

## Abstract

**Background:**

Understanding channel structures that lead to active sites or traverse the molecule is important in the study of molecular functions such as ion, ligand, and small molecule transport. Efficient methods for extracting, storing, and analyzing protein channels are required to support such studies. Further, there is a need for an integrated framework that supports computation of the channels, interactive exploration of their structure, and detailed visual analysis of their properties.

**Results:**

We describe a method for molecular channel extraction based on the alpha complex representation. The method computes geometrically feasible channels, stores both the volume occupied by the channel and its centerline in a unified representation, and reports significant channels. The representation also supports efficient computation of channel profiles that help understand channel properties. We describe methods for effective visualization of the channels and their profiles. These methods and the visual analysis framework are implemented in a software tool, ChExVis. We apply the method on a number of known channel containing proteins to extract pore features. Results from these experiments on several proteins show that ChExVis performance is comparable to, and in some cases, better than existing channel extraction techniques. Using several case studies, we demonstrate how ChExVis can be used to study channels, extract their properties and gain insights into molecular function.

**Conclusion:**

ChExVis supports the visual exploration of multiple channels together with their geometric and physico-chemical properties thereby enabling the understanding of the basic biology of transport through protein channels. The ChExVis web-server is freely available at http://vgl.serc.iisc.ernet.in/chexvis/. The web-server is supported on all modern browsers with latest Java plug-in.

**Electronic supplementary material:**

The online version of this article (doi:10.1186/s12859-015-0545-9) contains supplementary material, which is available to authorized users.

## Background

Protein channels are crucial for transport of ions, ligands, solvents and other macromolecules. Such channels occur in diverse systems such as enzymes where they play a role in navigating the ligand to a buried active site, or in channeling an intermediate through multiple entry and exit pathways, or in membrane proteins that are involved in the transport of small molecules, ions *etc*. The selectivity of the channels in permitting access to specific types of molecules and the micro-environment that it provides is crucial to the nature of the molecule that it transports [1]. Indeed, it is important to identify and study channels since mutations in residues lining the channel have resulted in channel dysfunctions. Such channelopathies have been associated with defective insulin secretion, diseases such as cystic fibrosis, epilepsy and kidney stone disease [2].

Geometrically, a *channel* is a pathway through the empty space within a molecule that connects an internal point and the molecular exterior [3]. A channel that passes through the molecule and connects two exterior points is called a *pore*. Other terms like *tunnel* and *molecular path* have also been used to refer to channels. However, we will consistently use the term channel to refer to both simple channels and pores. In this paper, we study the problem of efficient computation and effective visual exploration of channels in biomolecules. There is a need for an integrated framework that supports computation of the channels, interactive exploration of their structure, and detailed visual analysis of their properties. Although there exist tools that partly address this need, they either do not guarantee a robust computation of channels or they are found lacking in providing sufficient support for interactive visualization of channels and their properties. We aim to address these shortcomings, and develop a tool that uses sound mathematical theory for extraction of channels and also supports wide variety of intuitive and useful visualizations of channels and their properties.

### Related work

In recent years, numerous computational methods have been developed for detection and classification of empty spaces in proteins. Early techniques focused on finding cavities and pockets in molecules. These included grid-based approaches such as POCKET [4], LIGSITE [5] and VICE [6]. To overcome the inaccuracy of grid based methods, geometric and topological techniques were exploited to find cavities, more accurately, in software like CASTp [7], CAVER [8] and ProShape [9].

The problem of channel extraction^a^ was first addressed in HOLE [10]. The proposed solution involved splitting the molecule into slices along a user-specified vector and determining the largest empty sphere within each slice using simulated annealing. Similar approaches were used in other tools as well, most notably POREWALKER [11]. The idea of approximating the molecular space as a grid and determining channels by processing grid voxels has also been exploited in tools such as dxTuber [12], HOLLOW [13], 3V [14] and CHUNNEL [15]. Although this approach is computationally efficient, the accuracy depends on the grid resolution. Voronoi diagram based techniques avoid the need to choose approximate grid resolutions by directly representing balls and the space they occupy. However a key assumption is that the ion or molecule that traverses the channel may be represented by a ball. This approach is followed in MOLE [3,16], MOLAXIS [17], CAVER [8,18] and state of the art techniques developed by Lindow *et al.* [19,20] and Kim *et al.* [21]. MOLE uses pruned Voronoi diagram of atom centres for extracting channels. MOLAXIS and CAVER support differing atomic radii by approximating large atoms as a union of small balls with uniform radii. Lindow *et al.* compute the Voronoi diagram of spheres to further improve the geometric accuracy of channel centerlines. Our proposed channel extraction technique falls in the category of Voronoi diagram based methods. Different from the above, we use the alpha complex, which is based on the power diagram, to compute channels in biomolecules. The channels computed using this approach are guaranteed to be feasible. Various channel extraction techniques are studied and compared in a recent detailed review [22].

Many of the above-mentioned methods and software tools do not facilitate study of physico-chemical properties of the extracted channels, and focus only on computing geometric properties of the channel. After a recent update, MOLE [3] supports computation of some physico-chemical properties. However, there is a lot of scope for improvement in terms of how these properties are visualized and presented to the user.

### Contributions

We describe a method for channel extraction, based on the alpha complex, that ensures extraction of geometrically feasible channels. Our proposed alpha complex-based representation of channels allows the storage of both the volumetric region occupied by the channel and its centreline in a unified manner. We demonstrate the usefulness of this approach *via* multiple case studies describing the automatic extraction and ranking of transmembrane pores. To gain a deeper functional understanding of extracted channels, we propose the computation of channel profiles. The second contribution is the development of simple but information rich representations for effective visualizations of the channel and its profiles. The methods presented in this paper are implemented within a software tool called CHEXVIS, which is available as a web-server. Finally, CHEXVIS is compared with other channel extraction tools in terms of the supported features and evaluated based on the ability to identify known channels in a set of transmembrane proteins.

### Mathematical background

We briefly introduce the necessary geometric and topological background required to define and represent the structure of biomolecules. The reader may refer to the survey by Edelsbrunner [23] and a book on computational topology [24] for further details.

#### Voronoi diagram and Delaunay triangulation

**Figure 1:**
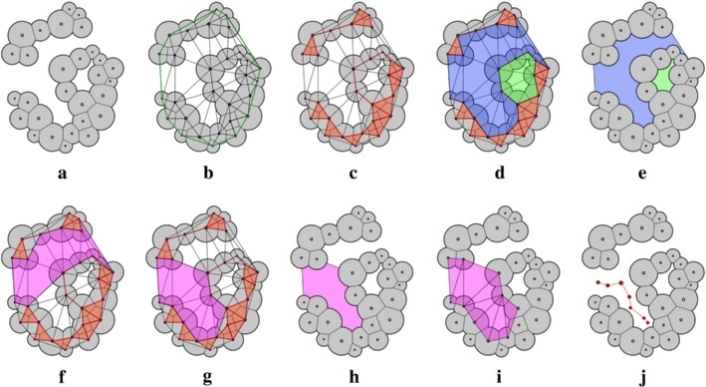
2D illustration of an alpha complex based representation of a molecule and the empty space within.**(a)** Union of disks (balls in 3D represent atoms) where the contribution from each disk is equal to its intersection with the corresponding Voronoi cell. **(b)** The weighted Delaunay triangulation of the disks and the convex hull (green). **(c)** Alpha complex at *α*=0, shown in red, is a subcomplex of the weighted Delaunay triangulation. **(d)** A cavity is a connected component of the complement of the alpha complex. A cavity with atleast one opening is a pocket (blue), while buried cavities are referred to as voids (green). **(e)** The empty space represented by the cavity triangles. **(f)** A channel is a simply connected subset of simplices of a pocket each of whose triangles has at most two neighbors and at least one boundary edge is a mouth edge. Here a pore (pink), a channel with two openings, is shown represented as a subset of the complement of the alpha complex. **(g)** A channel from the boundary to an interior point. **(h)** Underlying empty space of the channel. **(i)** Simplices of the complement of the alpha complex that represent the channel. **(j)** A path representation of the channel in which nodes are located at the centers of the orthogonal circle corresponding to each triangle and arcs connect nodes that correspond to neighbouring triangles.

Let $S \subseteq \mathbb {R}^{3}$ be a finite set of points in the 3D Euclidean space. The *Voronoi region* *V*_*p*_, of a point *p*∈*S* is the set of points in $\mathbb {R}^{3}$ that are closer to *p* than other points of *S*. The partitioning of $\mathbb {R}^{3}$ by Voronoi regions is called the *Voronoi diagram*. The *Delaunay triangulation* *D* of *S* is the dual of the Voronoi diagram representing the proximity relationship between Voronoi regions and partitions the convex hull of *S*. Vertices, edges, triangles, and tetrahedra of the Delaunay triangulation correspond to Voronoi regions, faces, edges, and vertices, respectively. The definitions of Voronoi diagram and Delaunay triangulation extend to points with associated weights. Let *w*(*p*) denote the weight of a point *p*∈*S*. The *power distance* between *p* and a point $x \in \mathbb {R}^{3}$ is equal to *π*_*p*_(*x*)=∥*x*−*p*∥^2^−*w*_*p*_. The *power diagram* and its dual the *weighted Delaunay triangulation* are defined as generalizations of the Voronoi diagram and Delaunay triangulation by replacing the Euclidean distance with the power distance [25], see Figure 1(b).

#### Molecular representation

Molecules are represented using the union of balls model, where each atom is represented as a ball whose radius is the *van der Waals* radius. A ball is represented by a weighted point whose weight equals the square of the ball radius. The power diagram restricted to the union of balls in a molecule can be used to represent the contribution from each atom towards the union of balls. The contribution from each atom *p* is equal to *B*_*p*_∩*V*_*p*_, the intersection between the ball corresponding to the atom and the weighted Voronoi cell of *p*. The corresponding dual structure is called the *dual complex*. The dual complex is a sub-complex of the weighted Delaunay triangulation and captures topological features such as voids. The complement of the dual complex provides a tetrahedral representation of the empty space in the molecule. Figure 1(c) shows the dual complex in red while Figure 1(d) illustrates how the complement of the dual complex captures empty regions in a molecule. Edelsbrunner *et al.* [26] considered a growth model, where they track changes in the dual complex corresponding to increasing ball radii. The growth parameter *α* corresponds to a radius equal to $\sqrt {{r_{p}^{2}} + \alpha }$ for a ball centred at *p* with radius *r*_*p*_. The dual complex corresponding to a set of balls that have grown by *α* is called the *alpha complex*.

As *α* varies from −*∞* to *∞*, topological features such as tunnels and voids appear in and disappear from the alpha complex. The importance of a topological feature is captured by the notion of *topological persistence* [24], which is equal to the length of interval of *α* for which the feature was present in the alpha complex.

## Methods

### Channel extraction

In this section, we describe a method of channel extraction based on alpha complex. As shown in Figure 1(c), the alpha complex and its complement partition the weighted Delaunay triangulation. For the case of *α*=0, the alpha complex represents the region covered by the atoms in the molecule. The complement of alpha complex, consisting of the remaining tetrahedra, triangles and edges of the weighted Delaunay triangulation, represents the empty space within the molecule.

The method proceeds as follows. First, a network of all geometrically feasible channels is constructed using the complement of alpha complex. Then, significant channels leading to active sites and important pores are identified within this network, depending on user-specified input. For identifying important pores, important end points are determined based on topological persistence [24]. A significant pore is reported for each pair of important end points. In the case of transmembrane proteins, a set of pores that traverse the membrane are identified and ranked in order of significance. Each extracted channel has a tetrahedral representation which accurately captures the volume occupied by the channel. The tetrahedral representation is exploited to efficiently compute channel profiles for every channel identified above. These profiles can be interactively visualized along with various 3D representations of the channel in the context of the biomolecular structure.

#### Channel network

**Figure 2:**
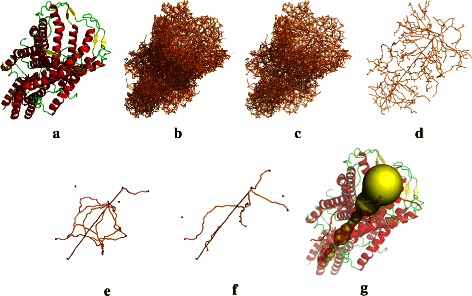
Channel extraction in a transmembrane protein.**(a)** Mechanosensitive Channel of Large Conductance (MscL, PDB id 2OAR). **(b)** The path network. **(c)** Widest path tree. **(d)** Pruned widest path tree. **(e)** Pores between top 10 boundary nodes in the network. **(f)** Transmembrane pores between top 5 interior and 5 exterior nodes. **(g)** Top ranked transmembrane pore shown using the skin surface (yellow). Other pores are shown for context.



All channels lie within the empty space of the molecule by definition. Therefore, we restrict our focus to those tetrahedra, triangles, and edges of the weighted Delaunay triangulation that do not belong to the alpha complex. We construct the dual graph of the complement to obtain a *channel network* in the molecule. The channel network is pruned by restricting to nodes that are accessible from the exterior because the empty space within voids is not accessible. The channel network is a subset of the power diagram of the set of atoms. A triangle in the empty space that lies at the interface of the molecular exterior and interior is called a *mouth* triangle, and represents an entry point into the molecule. The nodes in the channel network corresponding to tetrahedra incident on mouth triangles are called *boundary nodes*. Figure 2(b) shows the channel network for the transmembrane protein 2OAR. Algorithm ?? computes an annotated channel network, given the set of atoms in the molecule.

We also extract the *widest path tree* in the channel network by formulating it as a maximum spanning tree computation. This tree provides a good overview of the channel structures in the molecule, see Figure 2(c). The widest path tree can be further pruned so that it is a collection of paths between important nodes as shown in Figure 2(d). This pruned tree often includes all functional channels in the molecule. Detailed discussion on widest paths and the widest path tree is available in Additional file 1.

#### Significant channels

There exists multiple channels within the channel network between a given pair of nodes. We aim to identify a small set of potentially significant channels, one or more of which may correspond to biologically significant channel in the molecule. Short and wide channels are considered significant. We quantify significance by assigning a cost to each edge in the channel network equal to the ratio of the edge length to the minimum power distance along the edge. The power distance is an indicator of the local width of the channel. Next, given a set of endpoints, we compute the shortest paths between all pairs of endpoints using Dijkstra’s algorithm on the channel network. Dijkstra’s algorithm is executed multiple times on the network to obtain a set of important channels instead of a single most significant channel. After each iteration, weights on edges belonging to the detected channel are made high enough to ensure that subsequent iterations report edge disjoint channels. It should be noted that the naive strategy of executing Dijkstra’s algorithm only once and choosing top channels, usually yields a set of very similar channels. In the discussion below, we distinguish between the case when one of the end points is an active site and buried within the molecule and the case when both endpoints lie on the boundary of the molecule.

#### Channels to active sites

Enzymes contain buried active sites and it is often useful to compute and visualize channels leading to such sites. We accomplish this by first automatically determining a node from the channel network to represent the active site. Dijkstra’s shortest path algorithm is employed to compute the channel to the boundary. We use the Catalytic Site Atlas [27] and HETATM records of significant ligands in the PDB file to determine important sites within the given molecule. Both methods provide a set of atomic locations (*A*={*p*_1_,*p*_2_,…,*p*_*n*_}) from the active site. A representative node may be determined by computing the node closest to the centroid of the atoms that constitute the active site [3]. However, this approach may not work for large ligands where the centroid is typically far removed from the deepest point in the active site that interacts with the ligand. We assign a *depth* value to each node in the channel network via an iterative wave-front propagation algorithm that begins at boundary nodes and proceeds towards the interior. For each atom in the set *A*, we determine the closest node and hence construct the set *N*(*A*). We select the node *n*∈*N*(*A*) with the highest depth value as the representative node for the active site.

#### Extraction of pores

A channel both of whose endpoints are incident on mouth triangles is called a pore. Technically, any path between two boundary nodes of the channel network is a pore.

##### Important pores.

Computing significant channels between all pairs of boundary nodes to determine pores is a costly operation because the number of boundary nodes is usually large. We select a representative node from each mouth. Specifically, we locate the mouth triangle with the highest persistence [28] and choose the corresponding node as the representative. This mouth triangle roughly corresponds to the widest opening of the mouth. Our choice of the value of *α* is conservative. We determine *α*_*max*_∈[0,*∞*) at which the number of mouths attains a maximum value. The mouths are extracted at *α*_*max*_ and the triangle with the maximum persistence is chosen as the representative. The above procedure reduces the list of boundary nodes to a manageable size. Let *B*_*imp*_ denote the reduced list of nodes. This list is sorted in decreasing order of the persistence value of the tetrahedron corresponding to each node. Next, the top *k* boundary nodes are chosen from the list, where *k* is a user-defined parameter. Given *k*, channels between all possible pairs of the *k* nodes are automatically extracted as important pores in the molecule. Figure 2(e) shows the set of pores extracted in 2OAR for *k*=10.

##### Transmembrane pores.

Transmembrane pores are of special interest because of their functional importance. We use the OPM database [29] to classify nodes in *B*_*imp*_ into three sets *viz.* *B*_*in*_, *B*_*out*_, and *B*_*mem*_ corresponding to the nodes that lie inside, outside, or within the membrane, respectively. When OPM data is not available, the TMHMM utility [30] is used to classify the nodes. The pores are extracted such that one endpoint lies in *B*_*in*_ while the other lies in *B*_*out*_. Figure 2(f) shows the set of transmembrane pores. Let *TM* be the set of transmembrane pores extracted using this approach. We rank the pores in *TM* based on three criteria – length, bottleneck width, and straightness. Straight pores suggest highly regular and symmetric cavity structure around the channel centreline [11]. Straight pores are therefore preferable over winding pores in transmembrane proteins. The score *f*(*x*) assigned to a transmembrane pore *x*∈*T* *M* is defined as follows:

$$ f(x) = \frac{1}{3} \left(\frac{|x|}{\max_{y\in TM} |y|} + \frac{bn(x)}{\max_{y\in TM} bn(y)} + s(x)\right), $$

where |*x*| is the length of the pore *x*, *b* *n*(*x*) is the bottleneck width of the pore, and *s*(*x*) is a measure of how straight the pore is. The straightness is computed by calculating the deviation of path nodes from the best fit line. Let *S* denote the ordered list of uniformly distributed sample points along the path representation of the channel. The straightness term is given by the following expression:

$${}s(S) = \frac{\sum_{d=1}^{|S|/2}{\sum_{i=1}^{|S|-2d}{d \!\times \text{cos} \angle\left(S[i],S[\!i+d],S[i+2d]\right)}}} {{\sum_{d=1}^{|S|/2}}{\sum_{i=1}^{|S|-2d}{d}}} $$

The average curvature is computed at different scales from the set *S*. Curvature is approximated by the cosine of the angle formed by three equally separated sample points. The distance is varied to capture the straightness of the channel at different scales. The pore with the highest score *f* is reported as the best transmembrane pore. Figure 2(g) shows the best transmembrane channel detected in 2OAR. It should be noted that correctness of identified transmembrane pores is dependent on the accuracy of the information provided by the OPM database and TMHMM utility regarding the orientation and placement of protein with respect to the membrane.

#### Advantages of using alpha complex for channel extraction

Compared to earlier Voronoi based approaches [16,19], our proposed alpha complex based approach has the following advantages:

- The detected channels are guaranteed to be geometrically feasible. This follows from the definition of alpha complex. Delaunay triangulation based-approach, as used in Mole [16], can also provide tetrahedral representation for channels, but these channels are not guaranteed to be geometrically feasible.
- The tetrahedral representation of the volume occupied by the channel enables accurate computation of volumes and surface areas [31].
- The tetrahedral representation may be used for other computations such as those requiring finite element analysis.
- The tetrahedral representation is also used for computing channel profiles and hence plays a crucial role in our channel visualizations.
- Euclidean Voronoi diagram of spheres allows extraction of geometrically accurate channels [19,21], but a channel may not necessarily be represented as a set of tetrahedra using this approach.

### Channel visualization

#### Channel profiles

We propose the computation and visualization of *channel profiles*, 1D real-valued functions defined on the channel, with the aim of facilitating a quantitative analysis of the computed channels. Let $P \subset \mathbb {R}^{3}$ denote the centreline of a channel. A channel profile is a real-valued function defined on P. Let *p*_0_ be one end-point of the channel. The centreline can be parametrized using the geodesic distance from *p*_0_. So, the channel profiles are, indeed, real-valued functions defined on an interval [0,*d*], where *d* is the length of the channel.

##### Radius profile.

We compute the variation of the square root of power distance along the channel. We call this the radius profile because it is the radius of the orthogonal sphere to the three closest atoms. This profile provides a good estimate of the width of the channel, and is therefore useful for gaining information about the potential bottlenecks along the channel.

##### Electrostatic potential profile.

Electrostatic potential is computed for the whole molecule using the finite element method APBS [32]. The computed electrostatic field is available as a sample over a grid. We use tri-linear interpolation to determine the electrostatic potential values at points along the centerline. The use of default parameters for electrostatic potential computation and approximation along centerline may lead to a situation where this profile may provide a false impression of the charge distribution inside the channel. Therefore, the electro-static potential should be used for analysis with care.

##### Physico-chemical profiles.

We compute the average of different physico-chemical quantities over the four atoms of each tetrahedron in the channel. Important physico-chemical profiles include hydophobicity [33], charge, bulkiness, and secondary structure related profiles. We use ProtScale [34], which assigns a scalar value for each amino acid to represent a physico-chemical quantity.

##### Conservation profile.

Conservation captures the tendency of an amino acid residue to remain unchanged over long periods of evolution. Conserved residues may be of functional significance. We use the ConSurf server [35], which uses multiple sequence alignment over a large set of sequences, to obtain conservation score for all amino acid residues in the protein. The conservation score at each node is computed as an average of the conservation scores of the four amino acids incident on the corresponding tetrahedron.

#### Profile visualization

**Figure 3:**
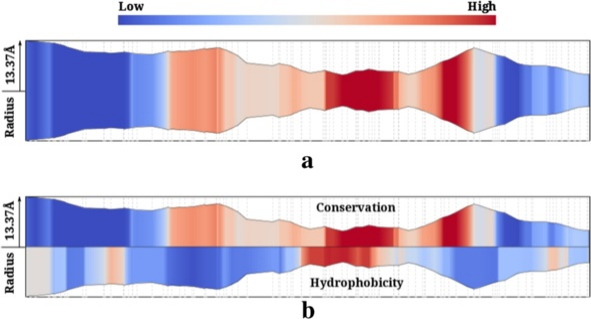
Channel profile visualization of the transmembrane pore in 2BG9.**(a)** Conservation profile shown as a color map over the radius profile, which in turn is shown as a symmetric graph plot. **(b)** Hydrophobicity and conservation profiles shown in a split visualization over the radius profile.

Channel profiles are visualized as a simple XY-plot that shows the variation of the property along the channel. To capture the correlation of the radius profile with a second channel profile, we compute a 2D projection of the channel using the radius profile. The second profile is shown using color-mapping^b^ within the resulting region, see Figure 3(a). This visualization may also be extended to display the correlation between the radius profile and two different profiles. The 2D projection is split into two, and each region displays a different channel profile using color-mapping as shown in Figure 3(b).

#### Channel visualization in 2D and 3D

**Figure 4:**
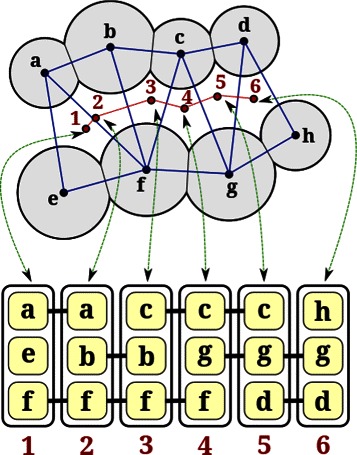
Illustration of 2D representation of a channel in a synthetic 2D example.**Top:** The molecule is shown as a set of disks (grey). The channel is shown as a path (red) and as a set of triangles (blue). In 2D, each node in the path corresponds to a triangle, while a path edge corresponds to an edge in the triangulation. Each disk is given an alphabetic label, while the nodes of the path are given numeric labels. **Bottom:** The proposed 2D visual metaphor of the channel is shown. Each vertical box denotes a node in the path (and thus denotes a triangle within the channel). The three small boxes within the node box denote the disks incident on the triangle corresponding to the particular node box. Also, consecutive nodes boxes are connected by two edges. These edges denote the edge shared by the consecutive triangles in the channel. For example, node boxes 3 and 4 denote triangles *cbf* and *cgf*, respectively. Their shared edge is *cf*, so the atom boxes corresponding to discs *c* and *f* are connected by edges. This representation naturally extends to the 3D case, with the only modification that each node box will denote a tetrahedron and will thus contain four atom boxes. Two consecutive node boxes will be connected by three edges denoting the common triangle shared by the adjacent tetrahedra.

**Figure 5:**
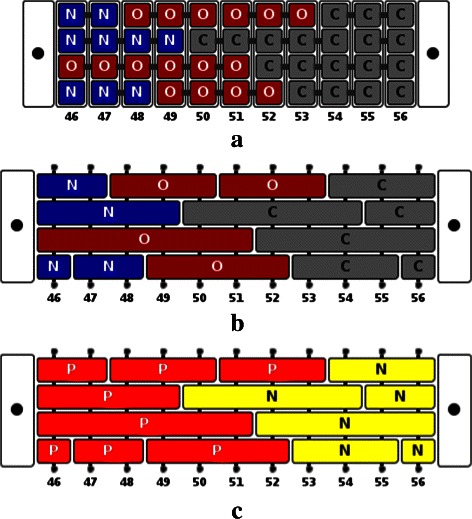
2D representation of a channel.**(a)** 2D box representation of the atoms lining a portion of the channel in 2BG9. **(b, c)** Boxes are merged and colored by atom type and polarity of residues. The numbers below the boxes are tetrahedron indices, which makes it clear that this visualization corresponds to a subset (tetrahedra 46-56) of the complete channel.

We design a novel visualization metaphor for channels based on tetrahedral representation of channels. A channel is visualized using the 2D projection. We use the correspondence between the channel network nodes and the weighted Delaunay triangulation tetrahedra, and between the channel network edges and the weighted Delaunay triangulation triangles to effectively visualize different properties along the channel. Each node is displayed as a *node box* containing four smaller *atom boxes*, which correspond to the four atoms at the vertices of the tetrahedron. The channel is shown as a sequence of node boxes. Adjacent node boxes are connected by three edges that correspond to the triangle shared by the two adjacent tetrahedra. Figure 4 illustrates this representation for a 2D example, while Figure 5 demonstrates this visual representation in the 3D case for a part of the transmembrane pore in 2BG9. Consecutive atom boxes are merged into one if they correspond to the same atom (Figure 5(b) and (c)). The power of this representation is realized when we treat each atom box as a place holder. Each box is given a label and a color. Appropriate choices of a colormap and labels for the boxes based on the physico-chemical properties results in a concise and effective visualization of the channel. This representation can be viewed along with profile visualization to obtain richer information about the channel. This visualization metaphor can be used for representing any channel represented as a set of tetrahedra. In particular, this visualization may also be used by tools like MOLE to show variation of physico-chemical properties across a channel.

The channel is also visualized in 3D within the context of the molecule. We support visualizing channels as tetrahedra, union of balls, or as a surface of the union of balls. The union of balls representation of the channel is obtained by computing the orthogonal sphere for each channel tetrahedron. The skin surface is a smooth surface that wraps around the given set of spheres [36]. We compute the skin surface for the union of balls representation of the channel. In addition to displaying the channel, we optionally show atoms or amino acid residues lining the channel. This may help the user to further inspect the physico-chemical properties of the channel. We also provide pymol scripts to facilitate download and visualization of the channel properties. The combined view of the channel and residue is pointing into the channel lumen coupled to their physico-chemical and biochemical properties and conservation in related proteins would be useful in assessing mutability of channel residues and their mapping on to the protein 3D structure.

## Results and discussion

The channel extraction and visualization methods presented in this paper are implemented into a software tool called CHEXVIS. A web-server [37] has been implemented and made freely available. The web-server is comparable to existing state-of-the-art channel extraction software in terms of functionality.

### Web server features

The CHEXVIS web-server supports submission of jobs by specifying the PDB ID or uploading a PDB file. Biological assemblies of proteins can also be uploaded. While the default values for the parameters often produce required results, the user may optionally tune multiple parameters. Further, the server also computes transmembrane pores similar to existing tools like POREWALKER that exclusively detect such channels. The unique and novel 2D representation of the channel enables a detailed analysis of its physico-chemical properties at a level of detail not supported by other existing software. The extracted channels and their properties are displayed within the browser using Java/JMol based applets. PyMOL [38] scripts for individual channels are also generated and may be downloaded for detailed off-line study. See Additional file 2 for typical output of the web-server.

### Comparison with other channel extraction tools

**Figure 6:**
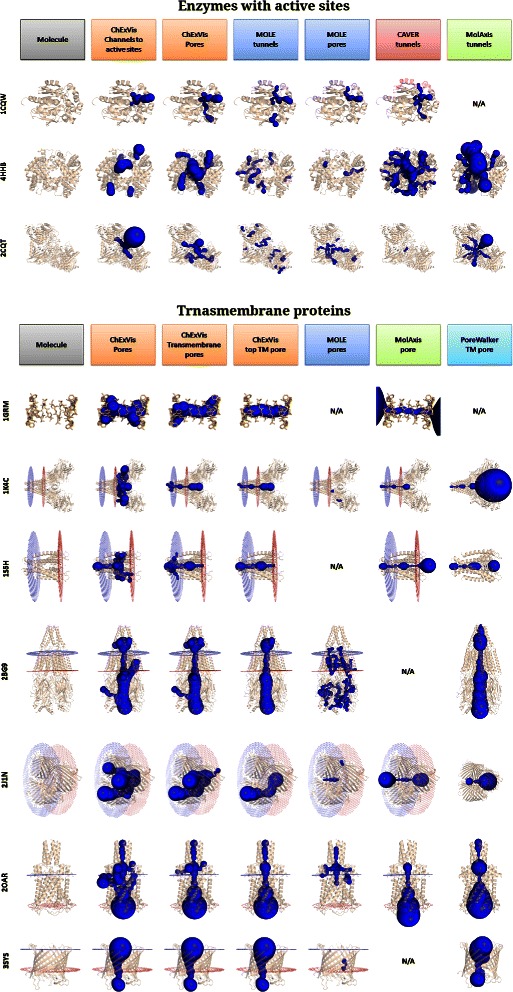
Comparison of channel extraction tools. Results obtained from different channel extraction tools, *viz.* CHEXVIS, MOLE, CAVER, MOLAXIS and POREWALKER, are summarized in a tabular form. On the top, results for some enzymes with active sites are shown while results for transmembrane are shown on the rows at the bottom. For complete comparison results, refer to Additional files 3 and 4.

We compare CHEXVIS web-server with four channel extraction tools *viz.* MOLE 2.0 [3], CAVER Analyst 1.0 [18], MOLAXIS [17] and POREWALKER [11]. Table 1 summarizes the features provided in these tools. As evident from the comparison table, the primary novelty of CHEXVIS is the advantage of extensive visual analysis of channels using channel profiles along with information-rich 2D representation of channels. However, we also claim that the proposed channel extraction method is significantly better than the other tools. To support our claim, we compared the results of these methods for a set of 29 diverse protein structures. The channels detected in the seven enzymes which are a part of our dataset, are summarized in Additional file 3, while pores extracted in 22 transmembrane structures by different methods are shown in Additional file 4. A subset of these results is shown in Figure 6.

**Table 1:** **Comparison of features supported in different channel extraction tools**

	**CHEXVIS**	**MOLE**	**CAVER**	**MOLAXIS**	**POREWALKER**
Computation of channels leading to internal molecular sites	Yes	Yes	Yes	Yes	No
Automatic suggestion of internal sites using CSA	Yes	Yes	Yes	No	No
Channels to multiple internal sites	Yes	Yes	Yes	No	No
Filtering of similar channels	No	Yes	Yes	No	No
Computation of pores	Yes	Yes	No	Limited	Only transmembrane pores
Computation of transmembrane pores	Yes	No	No	Limited	Yes
Multiple transmembrane pores	Yes	No	No	No	No
Ranking of pores	Yes	No	No	No	Not applicable
Computation of physico-chemical properties	Yes	Yes	No	No	No
Interactive visualization of physico-chemical properties	Yes	No	No	No	No
Conservation profile	Yes	No	No	No	No
User Interface	Web-server	Web-server and GUI	GUI	Web-server	Web-server
Interactive visualization of channels	Yes	Yes	Yes	No	No
Good default parameters	Yes	Yes	Yes	Limited	Yes
Computation speed	Reasonable	Fast	Reasonable	Reasonable	Slow
PyMOL export	Yes	Yes	Yes	No	No
PyMOL plugin	No	Yes	Yes	No	No

For comparison, we used the default parameters for channel extraction provided by the respective tool. CHEXVIS computes three types of outputs *viz.* channels leading to active sites, pores and transmembrane pores. MOLE computes channels leading to active sites^c^ as well as pores, while CAVER computes only the former. In addition to computing channels leading to a site in the molecule, MOLAXIS can also extract transmembrane pore if the axis and a constraint sphere are provided. POREWALKER computes a single transmembrane pore. We compare CHEXVIS channels with the channels computed by MOLE, CAVER and MOLAXIS. CHEXVIS pores are compared with MOLE pores. The top transmembrane pore computed by CHEXVIS is compared with the transmembrane pore computed by MOLAXIS and POREWALKER.

#### Summary of comparison

The qualitative comparison of results produced by different tools using default input parameters are summarized in Additional files 3 and 4. For the 22 transmembrane structures in our dataset, we also attempt a quantitative comparison of pores identified by these tools, by reporting the number of structures in which biologically significant pores are correctly identified. The biologically significant pores (the ground truth) are determined based on prior studies reported elsewhere in literature.

##### MOLE.

MOLE provides excellent stand-alone and web-based interactive interface for extraction of multiple channels. MOLE utilizes the CSA database [27] and HETATM records to automatically identify catalytic sites in the protein. This facility is also supported by the CHEXVIS web-server. As evident from results in Additional file 3, compared to CHEXVIS, MOLE reports more tunnels leading to active sites. This is due to the fact that CHEXVIS reports a single best channel from each active site, while MOLE extracts multiple tunnels and clusters the extracted channels to reduce the number of reported channels. The option to extract multiple channels from a single point of origin is available in a stand-alone version of CHEXVIS and will be included into the web-server soon.

**Figure 7:**
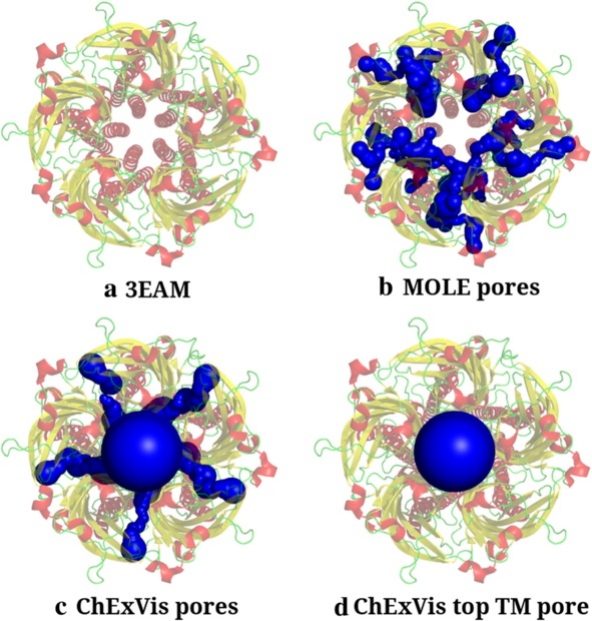
Comparison of pores extracted by MOLE and CHEXVIS in 3EAM.**(a)** Cartoon representation of the transmembrane protein 3EAM. The 3D view is such that the transmembrane pore is perpendicular to the plane of the page. **(b)** The pores extracted by MOLE in this structure. MOLE identifies a few side channels going from central pore to outside, but it fails to identify the transmembrane pore. **(c)** On the other hand, CHEXVIS identifies the main transmembrane pore as well as some side channels. **(d)** Also, the top transmembrane pore suggested by CHEXVIS is verified to be the correct transmembrane pore in this structure [39].

**Figure 8:**
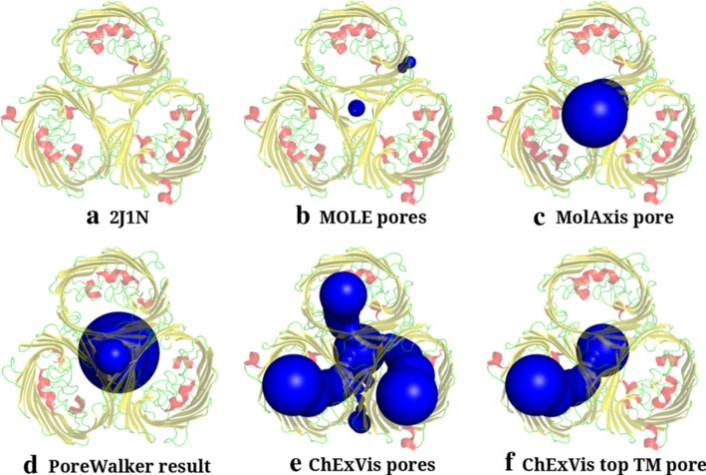
Comparison of pores extracted by MOLE, MOLAXIS, POREWALKER and CHEXVIS in 2J1N.**(a)** Cartoon representation of the transmembrane protein 2J1N. This structure consists of three beta-barrel subunits going across the cell membrane. The figure shows top view of the protein such that cell membrane is parallel to the plane of the page. **(b)** MOLE identifies two pores in this structure, both of them are not correct transmembrane pore passing through beta-barrels. MOLE wrongly identifies the narrow space between the three units as a transmembrane pore. **(c)** MOLAXIS also identifies a pore going through space between three subunits. This maybe due to the specified input parameters. **(d)** POREWALKER also fails to identify the correct transmembrane pores in this structure. It identifies the empty space between three subunits as the only transmembrane pore. By design, POREWALKER would not have been able to identify all the three transmembrane pores as it extracts only one transmembrane pore. **(e)** CHEXVIS is able to correctly identify transmembrane pores through all the three subunits, using default parameters for finding pores. **(f)** Unlike other tools which identify pore through the space between subunits, the top transmembrane pore identified by CHEXVIS is one of the pore passing through a beta-barrel subunit.

However, MOLE does not support extraction of transmembrane pores. We used its pore extraction option to determine pores and compared the results against pores extracted by CHEXVIS. Among the 22 transmembrane proteins, using default parameters MOLE fails to identify correct transmembrane pore in 17 structures. In comparison, CHEXVIS identifies correct transmembrane pores in 19 structures and partially correct pores in the remaining 3 structures (see Additional file 4). Figure 7 compares the results obtained by MOLE and CHEXVIS in the PDB structure 3EAM. MOLE fails to identify the long and wide pore going across the membrane, and instead identifies a few side channels. CHEXVIS, on the other hand, correctly identifies the biologically significant pore [39] as the top ranked transmembrane pore. It also identifies some of the side channels identified by MOLE. Figure 8 demonstrates another example where MOLE does not report correct transmembrane pores in 2J1N.

##### CAVER.

CAVER is a command-line based channel extraction tool. A commercial GUI front-end of this tool was released recently as CAVER Analyst 1.0 [18]. We used the evaluation version of CAVER Analyst 1.0 for the experiments. CAVER Analyst supports automatic identification of active sites using CSA database. However, the number of origin points in a particular run is limited to one. The tunnels extracted by CAVER are reported in Additional file 3. Qualitatively, the results reported by CAVER are not better than those reported by CHEXVIS. CAVER was not used for comparison of pores extracted in transmembrane proteins, as it does not provide special support for extraction of pores in general and specifically transmembrane pores.

##### MOLAXIS.

MOLAXIS uses a Voronoi-based approach for channel detection. It automatically uses the center of largest cavity as the origin point of the channel. This is a useful heuristic but this single point may not correspond to an active site. Further, channels leading to multiple sites cannot be extracted in a single run. As shown in Additional file 3, MOLAXIS fails to identify channels leading to active sites in two of the seven enzymes. MOLAXIS also provides an option of extracting a transmembrane pore, but it requires specification of the pore axis and a guiding sphere, which requires prior analysis of the protein and smart inputs by the user. As a result, it is difficult to identify transmembrane pores using MOLAXIS. When reasonable default values are chosen for pore axis and a guiding sphere, we observed that MOLAXIS computes correct transmembrane pores in 12 out of 22 transmembrane proteins (see Additional file 4). In cases of success, the extracted transmembrane pore mostly agrees with the top transmembrane pore detected by CHEXVIS.

##### POREWALKER.

POREWALKER is the state-of-the-art tool specially designed for extraction of transmembrane pores. Given a transmembrane protein, POREWALKER reports the best transmembrane pore and residues lining that pore. This tool correctly identified biologically significant pore in 17 out of 22 structures while partially correct pores in 3 other structures (see Additional file 4). However, it was unsuccessful in identifying correct pores in two structures in our dataset. One such example is reported in detail in Figure 8. Here, instead of identifying any of the three biologically significant pores through the beta-barrels, POREWALKER incorrectly identifies the empty space between three units as the most significant transmembrane pore. Moreover, POREWALKER is designed to report only one transmembrane pore. So, in cases such as 2J1N (Figure 8), POREWALKER does not report all three transmembrane pores, which are equally important. CHEXVIS overcomes this problem by reporting multiple transmembrane pores, and in this example correctly identifies all three pores as shown in Figure 8(e).

### Applications to the analysis of channels in proteins

The 2D and 3D visualization of the channel, coupled to a display of its various properties of the channel through profile plots, is an important feature and advancement over existing channel visualization tools. The biochemical layout of the channel lining residues and capture of their physico-chemical properties such as radius, volume and solvent accessibility are useful in appreciating the channel type and its lining residues. A novel feature is the inclusion of conservation scores derived through comparison of residue propensities in related channels using the CONSURF web-server [35]. This is useful in evaluations of the observed mutability amongst closely related homologues and in evaluating residues that may be substituted in experimental mutagenesis, or in guiding the decisions on the substitutions that are likely to be tolerated. Further, the profile layouts help evaluate the importance and contribution of the observed amino acids at various positions, to appreciate interactions that are observed in the channel and are crucial to its function. With the following examples we describe some applications of CHEXVIS in the detection of channels and its use in appreciating the function of the channel at various levels of detail.

#### Comparison of open and closed states of the protein

##### Pentameric ligand-gated ion channels.

**Figure 9:**
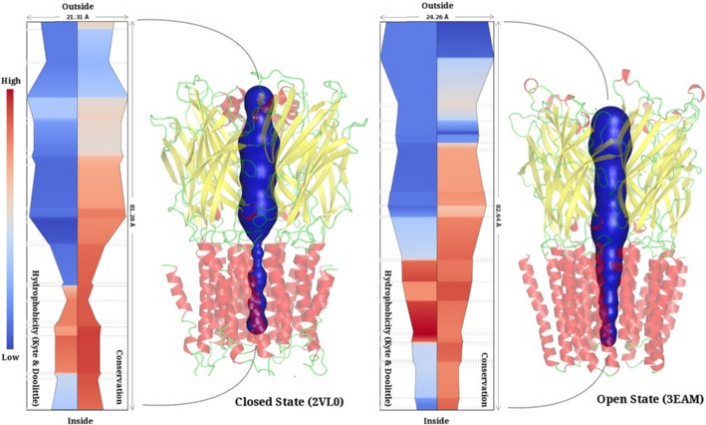
The pentameric ligand-gated ion channels extracted in closed and open conformations. The channels are wide and hydrophilic in extra-cellular region which is less conserved. The constricted transmembrane spanning helical channel section is hydrophobic and highly conserved. The bottleneck radius is 2.21Å in closed configuration while it is 5.12Å in open state.

Pentameric ligand-gated ion channels constitute a large family of ionotropic channels that are ubiquitously represented and fairly conserved in the animal kingdom [40]. The 3D structures of a number of such channels were submitted to CHEXVIS to derive pore features. A homo-pentameric organization that is constituted by a highly conserved extracellular domain folded as a beta-sandwich and a helical transmembrane domain [41], is captured in all the queried proteins. We compared profile views of the ligand-gated ion channel in its closed (2VL0:ELIC) and open states (3EAM:GLIC), to determine if CHEXVIS could capture the similarities and differences in these states through analysis of features such as: a) the changes in the diameter of the channel, b) accessible solvent area/volume of the channel, c) residues lining the channel, and, d) physicochemical properties of residues lining the channel. As seen in the profile views, in both states, pore-interfacing residues from all the five chains lie in fairly equivalent positions. The 2D profile plots of the residue contributions to the channel show that the side chain atoms of the channel-lining residues point into the channel lumen (see Additional files 5 and 6). Differences in the open (3EAM:GLIC) and non-conducting closed conformation of the channel (2VL0:ELIC) include a constriction lined by hydrophobic residues, possibly contributing to ion-selectivity and corresponding to a bottle-neck radius of 2.21Å while this is 5.12Å in the open state (3EAM:GLIC) [42]. Labelled view of the profiles show that F246 in 2VL0 lies at the narrowest region of the channel, as reported elsewhere [43]. The open and closed states of the channel also show marked differences in their relative solvent accessibility, as reported through their DSSP accessibility scores. The pore opening in 3EAM is lined predominantly by hydrophilic residues and subsequently followed by hydrophobic residues (Figure 9), in line with the structural findings of a 12Å wide extracellular hydrophobic vestibule and a funnel shaped transmembrane-spanning helical region. The conducting conformation in 3EAM, where the hydrophobic constriction has opened to an aqueous funnel-shaped channel is also captured [44].

**Figure 10:**
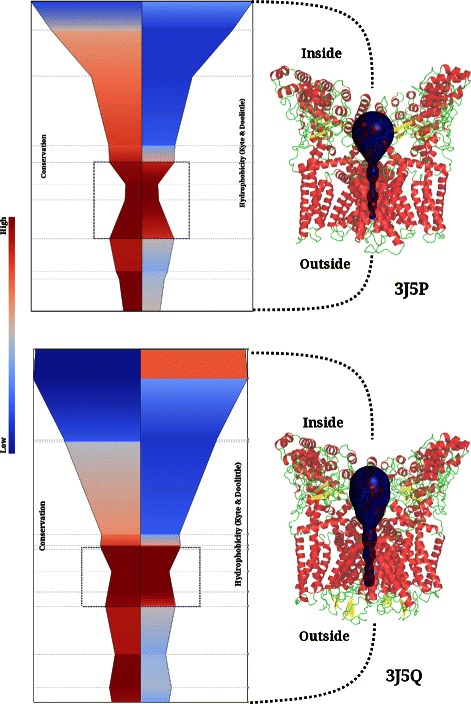
Transient Receptor Potential (TRP) channels extracted in closed and open state. The constricted region of the channel (highlighted by rectangles) is both highly conserved and hydrophobic. The bottleneck radius is 2.6Å in closed configuration while it is 4.66Å in open state.

CHEXVIS also correctly identifies the tetrameric assembly of the Transient Receptor Potential (TRP) channel and lists residues lying on the outer pore, central canal, and selectivity filter. As seen in Figure 10, the entry to the channel is a wide funnel-shaped pore in both the closed (3J5P) and open (3J5Q) states of the channel. The residue-label view shows that the narrowest region of the channel is lined by residues G683, I679 and Y671 with their side chains pointing into the channel. I679, the bulkiest residue in the lower gate of the channel, from each subunit comes together to form the most constricted point in the gate that seals off further access to the channel. In line with structural findings, its conservation scores shows that it is conserved in all homologues [45]. The radius of the hydrophobic seal in the lower gate changes from 2.6Å to 4.7Å in the two structures. Clearly, although lining residues do not change remarkably, side chains re-orient as a result of gating, resulting in a wider pore in the open state of 3J5Q (See Additional files 7 and 8 for detailed comparison of channels).

#### Comparison of channels exhibiting wide substrate specificities

**Figure 11:**
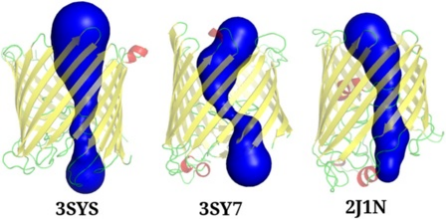
Outer Membrane (OM) carboxylate channels in structures 3SYS and 3SY7. These channels are narrower compared to channel porin (2J1N) indicating that they are more selective.

**Figure 12:**
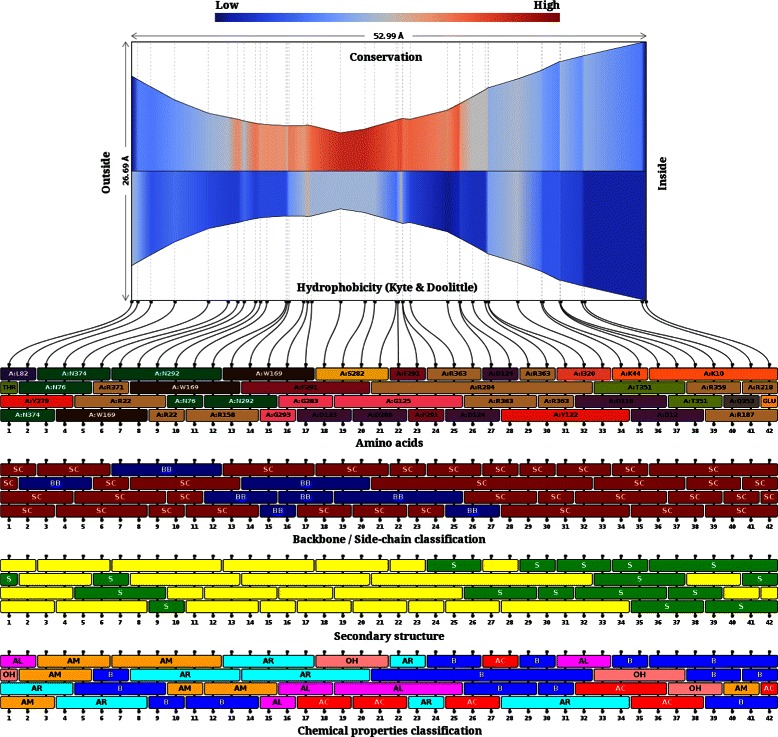
Properties of membrane carboxylate channel in 3SYS. The 2D profile is coloured by conservation and hydrophobicity of residues. It can be observed that the channel constriction is more conserved than the rest of the channel. According to box representation of first row, the amino acids lining the channel shows higher proportion of Arginine. Most of the residue side chains point towards the channel as can be concluded by many red boxes in the second row. The third row shows that channel is surrounded by loops (coloured yellow), specially at the constriction. Although some residues at channel end points belong to outer beta-barrel structure. Lastly, the fourth row shows the chemical properties of the residues lining the channel. The basic residues which play an important role in the function of this channel are correctly identified by ChExVis. They are represented as dark blue boxes.

The outer membrane proteins of several gram negative bacteria enable uptake of nutrients either through non-specific channels/porins and therefore, at high substrate concentrations, or in a substrate-specific manner through outer membrane carboxylic acid channels (Occ family). Since the Occ channel proteins are dedicated to the uptake of wide variety of substrates, we derived the pore features for different members of this family (3SY7, 3SY9, 3SYB, 3SYS, 3SZD, 3SZV, 3T0S, 3T20 and 3T24) and compared them to determine if the visual capture of the channel properties through 2D and 3D views could distinguish their various sub-types. Comparisons of the two OM channels, OccD1 (3SY7) and OccK1 (3SYS) shows that both channels form monomeric barrels around a central channel that is constricted at a region lined predominantly by basic residues such as arginine and lysine (see Figures 11, 12 and Additional file 9). Four of the seven residues forming the basic ladder in this class of proteins (OccK1:3SYS) are captured by CHEXVIS while projections of the channel neighbourhood capture other basic residues that are typical of the family (R22, R126, R158, R324, R306, R381 and R363). The plots also show that the constricted regions are contributed by loops and lined by residues that are poorly conserved in related homologues except for regions in the immediate vicinity of the constriction. Pore size can dictate the size of the substrate that can pass through the channel [46]. The differences in the pore size of the two amilies, with larger pore size observed in OccK family members to recognize a wider range of mono-cyclic substrates such as glucoronate and aromatic amino acids is correctly captured [47]. Contrastingly, the OccD family proteins have smaller pores that are highly specific and prefer smaller amino acids such as arginine. We also compared the Occ channels with porins (2J1N), which are non-specific channels with larger pores, and observe that the pore size of Occ family protein is indeed small and selective for small molecules [47]. We anticipate that visualization tools such as CHEXVIS that capture various views and properties of the residues lining the channel will add to knowledge on channel specificity and residue layout within the pore and a better appreciation of the differences between seemingly related molecules.

#### Comparison of channels in homologues and the impact of residue mutations on channel properties

**Figure 13:**
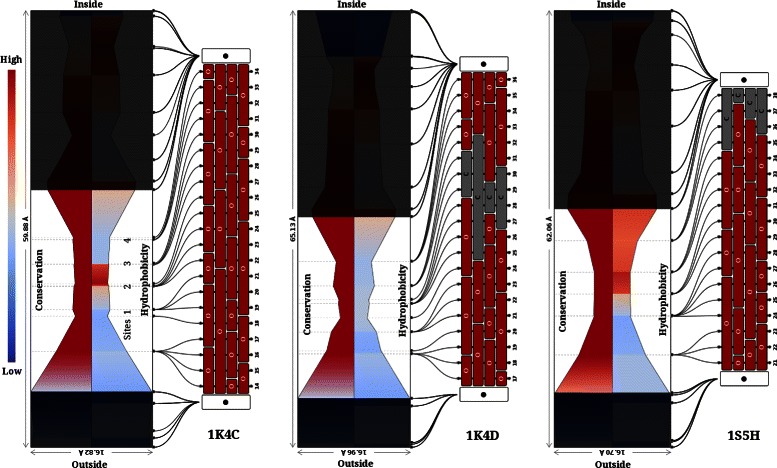
The KcsA potassium channels extracted in structures 1K4C, 1K4D and 1S5H. On left, channel extracted in 1K4C (high K^+^ concentration) is shown. The channel has four highly conserved K^+^ sites which are surrounded by carbonyl Oxygens as shown in the profile shown. The channel closes in low K^+^ concentration (1K4D) as captured by next profile. The channel in this structure is more constricted and one of the site is surrounded by Carbon atoms instead of carbonyl Oxygens. Lastly, on right a mutant channel (1S5H) is shown which has reduced K^+^ conduction capability. This is attributed to replacement of Oxygens with Carbon atoms at crucial site no. 4, which is correctly captured by CHEXVIS in the profile shown.

Voltage-activated cation channels are membrane proteins that selectively conduct K^+^, Na^+^ and Ca^2+^ ions in response to changes in membrane voltage. The KcsA potassium channel contains two trans-membrane segments and a P-loop signature that harbours a selectivity filter, which selects for the conductance of K^+^ over Na^+^ ions. Residues with a role as the filter from the four subunits, project into the channel centre to form a narrow pore. The K^+^ ion-binding sites formed by residues G79, Y78, G77, V76 and T75, that form the ion binding site and square anti-prism around K^+^ [48], are consistently identified by ChExVis in all the fourteen KcsA channels in the dataset (see Additional files 10 and 11). Although the path into the cavity is not identified by the method, the rest of the channel and a large central cavity (≈ 10Å), on the intracellular side, is correctly defined. Further, comparison of the structures at low (1K4D) and high salt (1K4C) concentrations shows that the conformation of the channel changes and moves from a closed to open state with increasing salt concentration (Figure 13). G77 contributes to channel constriction in 1K4D with a channel radius of 2.01Å. In 1K4C, the open state, V76 also lines the channel and coordinates the K^+^ ion through its carbonyl oxygen. Replacement of T75 with C75 in the mutant channel [49] also results in a widening of the channel, changing the bottle-neck radius from 2.26Å to 2.55Å. The mutant channel has low K^+^ conductivity because of the absence of crucial oxygen atoms. This minor but critical change is captured by CHEXVIS visualizations (Figure 13, see Additional file 11).

## Conclusions

We developed a new method for the extraction, visualization, and visual exploration of channels in biomolecules through a software tool, CHEXVIS, that is available as a web server. The method is automated and accepts 3D co-ordinates of the structure or its PDB-ID as input. Channel location in the protein may be guided using bound ligand information, CSA, or be specified by the user. Here, channels are represented as a set of connected tetrahedra derived from the alpha complex. This representation enables efficient computation and interactive visualization of the channels together with different geometric and physico-chemical profiles.

The availability of visualization tools such as CHEXVIS that can identify multiple channels will be useful to understand the properties of the multiple channels detected in the protein family of interest and the multiple routes that might be available for regulation of protein function in the family. The method is shown to perform well using a number of examples. As with all channel visualisation tools, manual intervention and intuition are vital to assess the importance of the reported channels. Examples of its application in understanding the biological function of the protein at various levels such as in the comparison of open and closed states of the protein, or in appreciating the wide substrate-specificities of the channel or comparison of channels with homologues have already been shown here. Further, the method lists the top channels for the protein and also reports all channels observed in the protein. Such studies coupled with information on conservation within the protein family and mutability of residues lining the channel will further the understanding of the basic biology of transport through membrane proteins.

## Endnotes

^a^ We use the term *channel extraction* because we view the problem as a special case of the *feature extraction* problem. The features of interest are channels and the data is a biomolecule represented in PDB format.

^b^ We use blue to red diverging color map, where blue corresponds to low values while red represents high values.

^c^ MOLE and CAVER use the term *tunnel* to refer to a channel that leads to a site inside the molecule, *i.e.*, tunnels have one end point incident on a group of atoms buried inside the molecule while the other end point is close to the molecular exterior.

## Additional files

Additional file 1
**Detailed description of widest channels and widest channel tree.** This document describes an alternate notion of best paths which considers only the width of the channel as an optimization criteria.

Additional file 2
**A typical output of** CHEXVIS **web-server.** Screen-shot of output generated by ChExVis for PDB-id 2BG9. The output page has 3 main regions (colored boxes shown only in this illustration). On top left (red box), the automatically computed channels extracted by ChExVis are listed along with their properties in tabular form. The user can select one of the rows of these tables to view that particular channel. On top right (blue box), the 3D view of the currently selected channel is shown in the context of the protein within JMol browser plug-in. At the bottom (green box), the 2D representation of the channel is shown in a Java applet called ChExVis properties viewer. Both the 3D and 2D views of the channel are highly interactive and provide rich visualization features. The views are linked to tables listing the channels. Selecting a different channel immediately updates the 2D and 3D views.

Additional file 3
**Comparison of channel extraction tools for enzymes.** The results of extraction of channels in a select set of enzymes using different tools *viz.* ChExVis, Mole, Caver and MolAxis.

Additional file 4
**Comparison of channel extraction tools for transmembrane proteins.** The results of extraction of pores in transmembrane proteins using different tools *viz.* ChExVis, Mole, MolAxis and PoreWalker.

Additional file 5
**Properties of pentameric ligand-gated ion channel in the open structure 3EAM.** The plots show residues from all five chains that interface with the pore in equivalent positions. The wall of the pore is lined primarily by the side chain atoms of various residues. The view shows that pore interior from positions 32-58 is least accessible while the positions 1-30 are solvent accessible. Channel positions 34-47 lined by residues I240, A237, I233, S230 are hydrophobic and lie on alpha-helices that correspond to the helical wall of the transmembrane spanning regions in the protein.

Additional file 6
**Properties of pentameric ligand-gated ion channel in the closed structure 2VL0.** Compared to channel in open state (3EAM), the channel in 2VL0 is much more constricted at the transmembrane helical region. There is also a drastic reduction in solvent accessibility of this region from 40Å^2^ to 10Å^2^. Some changes in chemical properties of the the lining residues are also observable in closed conformation, as N250 and F246 protrude towards the pore around constriction as against I240 and I233 in open state. Also, in these channels the extra-cellular tunnel lined by hydrophilic residues is not very conserved, while the narrow selective region lying in transmembrane region is highly conserved.

Additional file 7
**Properties of transient receptor potential channel in the closed structure 3J5P.** In this structure, the radius of the channel is only 2.6Å at the narrowest point. The 2D profile is coloured by conservation and hydrophobicity, red color denoting high values while blue denotes low values. It can be observed the constricted region is highly conserved and hydrophobic. The next five rows of box representations show the properties of amino-acids lining the channel. From this data, it can be concluded that I679 lies at the narrowest point of the channel. Further I679 is a bulky residue with side-chain protruding towards the channel and it has low accessibility. According to third row, most of the residues lining the channel are part of alpha-helices and small helical turns towards the end.

Additional file 8
**Properties of transient receptor potential channel in the open structure 3J5Q.** In this structure, the radius of the channel is 4.66Å at the narrowest point, which is substantial improvement over closed structure bottleneck radius of 2.6Å. The 2D profile is coloured by conservation and hydrophobicity, revealing that the constricted region is highly conserved and hydrophobic. It can be seen most of residues lining the channel are same as that in 3J5P. Again I679 is an important residue lying at the constriction point. It is important to observe that accessibility of the channel residues in 3J5Q is higher than in closed state i.e. 3J5P. Specially, accessibility of I679 goes up from 16Å^2^ to 65Å^2^.

Additional file 9
**Comparison of three outer membrane carboxylate channels.** The 2D representations of channels from top to bottom correspond to 3SYS, 3SY7 and 2J1N respectively. The split-profiles are coloured by conservation and hydrophobicity of residues, red denoting higher values while blue denotes low values. The amino acids lining these channels are also shown using box representation. A high proportion of basic residue Arginine (coloured as light brown box) in the channel neighbourhood can be clearly observed.

Additional file 10
**KcsA channels.** KcsA channels extracted in different structures by ChExVis. The PDB ID of the structures are shown below each figure.

Additional file 11
**Comparison of three transmembrane KcsA channels.** From top to bottom, properties of channels in 1K4C, 1K4D and mutant 1S5H are shown. The mutation of T75 to C75 is correctly captured by ChExVis as highlighted by a box in 1S5H lining residues. We can clearly observe a few green boxes change to orange in the mutated channel. This mutation also affects the physico-chemical properties of the channel as shown subsequent rows of the above figure. For example, chemical property, hydrophobicity and polarity exhibit clear change. Moreover, in the mutated structure there are fewer Oxygen lining the channel, which are critical for functionality of this transmembrane channel. Also, the channel becomes constricted in lower concentration of K^+^ ions as captured in 1K4D channel profile. In this configuration, the critical Oxygen atoms are replaced by Carbon atoms as highlighted by a box.
