# Supplementary material for: CHEXVIS: a tool for molecular channel extraction and visualization
Source: BMC Bioinformatics. 2015 Apr 16;16:119. doi: 10.1186/s12859-015-0545-9 (PMC4411761; doi:10.1186/s12859-015-0545-9)
Supplement: Additional file 1 — Detailed description of widest channels and widest channel tree. This document describes an alternate notion of best paths which considers only the width of the channel as an optimization criteria. [file 12859_2015_545_MOESM1_ESM.pdf]

S1 Widest Channels

As discussed in main manuscript, we use a heuristic to determine short and wide channels between two nodes in the network. As a variant we focus only on the width criterion to compute the channel with the maximum bottleneck width between two nodes. Edges in the channel network are assigned a cost equal to the minimum power distance along the edge. First, the maximum spanning tree T is computed for this weighted network. Next, the widest path between a pair of nodes is computed using a simple depth first search traversal in T. This procedure is much faster than applying Dijkstra's algorithm for computing the best paths. An important property that they satisfy is that given two nodes, u and v, that lie on the widest path P, the widest path between u and v is also contained in P. In other words, these paths are both locally and globally wide.

Widest escape channels

Given a source node in the network, we can compute all the widest channels leading to the boundary nodes using a depth first search traversal. Multiple escape channels with maximum width may exist. We choose the shortest among them as the *widest escape channel.* These widest escape channels are an alternative means of computing channels leading to active sites.

Widest channel tree

The maximum spanning tree of the channel network is called the *widest channel tree (forest)* of the molecule. This tree also provides a better overview of the channel structures in the molecule compared to the original channel network, as can be observed by comparing Figures 2(b) and (c). The tree can be further pruned to the widest channels between only the important boundary nodes B_{imp}. This pruned tree is shown in Figure 2(d). The reduced tree provides a good overview of the channels in the molecule and its computation is efficient.

Shortest and widest channels

The widest channels computed as described above may result in long winding channels. With further processing, we can find channels that have the same bottleneck width as the widest channel but are shorter in length. We first compute the maximum bottleneck width w_{max} using the maximum spanning tree. Then the original channel network is pruned based on w_{max}, where all the edges with width less than w_{max} are deleted. Let $PN = (N, E)$ be the original channel network. The pruned channel network PN' is computed as

$$PN' = (N, E' = \{e|e \in E \text{ and width}(e) \geq w_{max}\})$$

Now, the cost of each edge in PN' is set to be equal to its length. The shortest path between two nodes in PN' is computed using Dijkstra's shortest path algorithm. This shortest path is guaranteed to have the maximum bottleneck width.
