# Supplementary figures and images for: CHEXVIS: a tool for molecular channel extraction and visualization
Source: BMC Bioinformatics. 2015 Apr 16;16:119. doi: 10.1186/s12859-015-0545-9 (PMC4411761; doi:10.1186/s12859-015-0545-9)

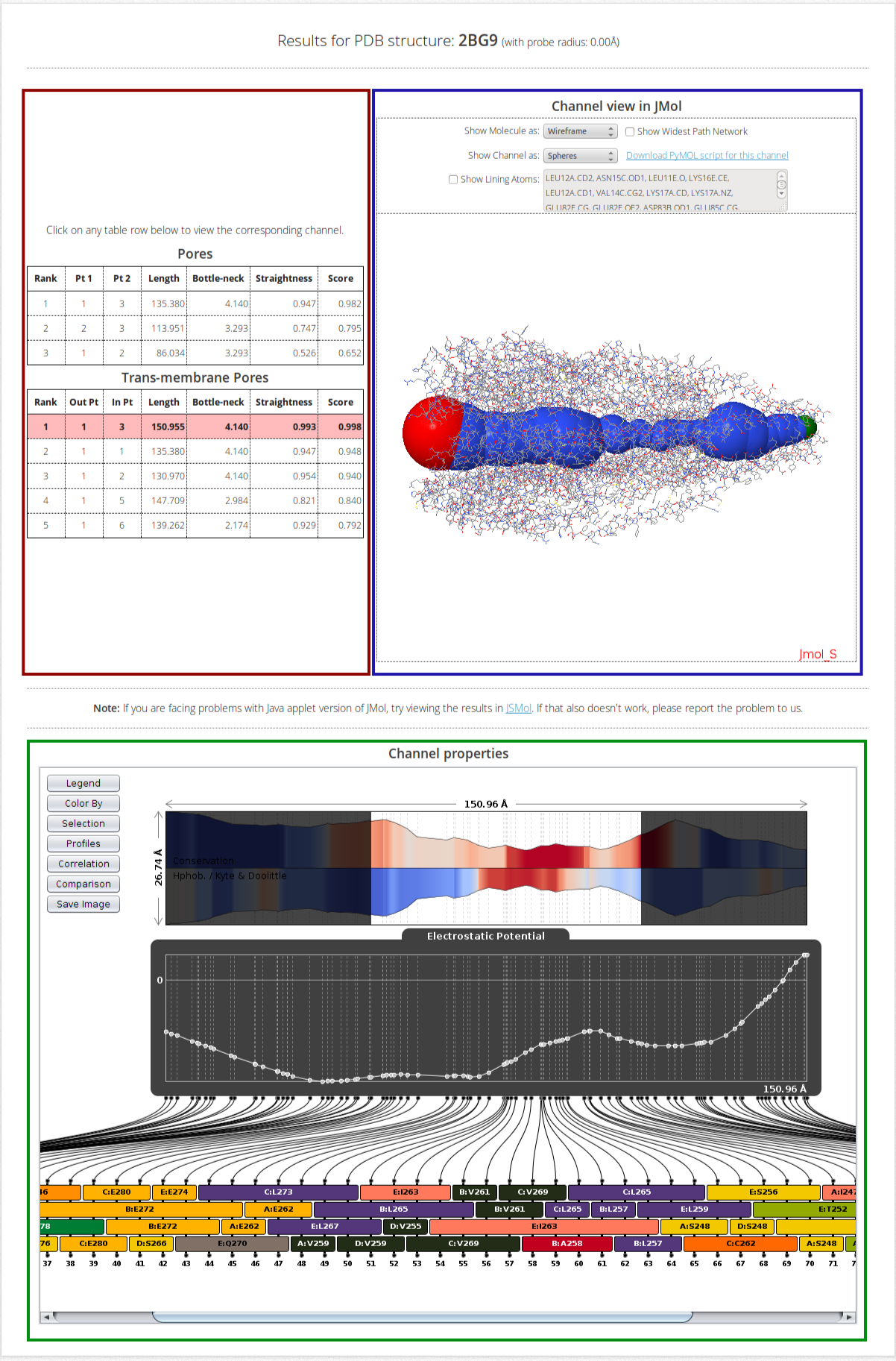

Supplement: Additional file 2 — A typical output of CHEXVIS web-server. Screen-shot of output generated by ChExVis for PDB-id 2BG9. The output page has 3 main regions (colored boxes shown only in this illustration). On top left (red box), the automatically computed channels extracted by ChExVis are listed along with their properties in tabular form. The user can select one of the rows of these tables to view that particular channel. On top right (blue box), the 3D view of the currently selected channel is shown in the context of the protein within JMol browser plug-in. At the bottom (green box), the 2D representation of the channel is shown in a Java applet called ChExVis properties viewer. Both the 3D and 2D views of the channel are highly interactive and provide rich visualization features. The views are linked to tables listing the channels. Selecting a different channel immediately updates the 2D and 3D views. [file 12859_2015_545_MOESM2_ESM.png]

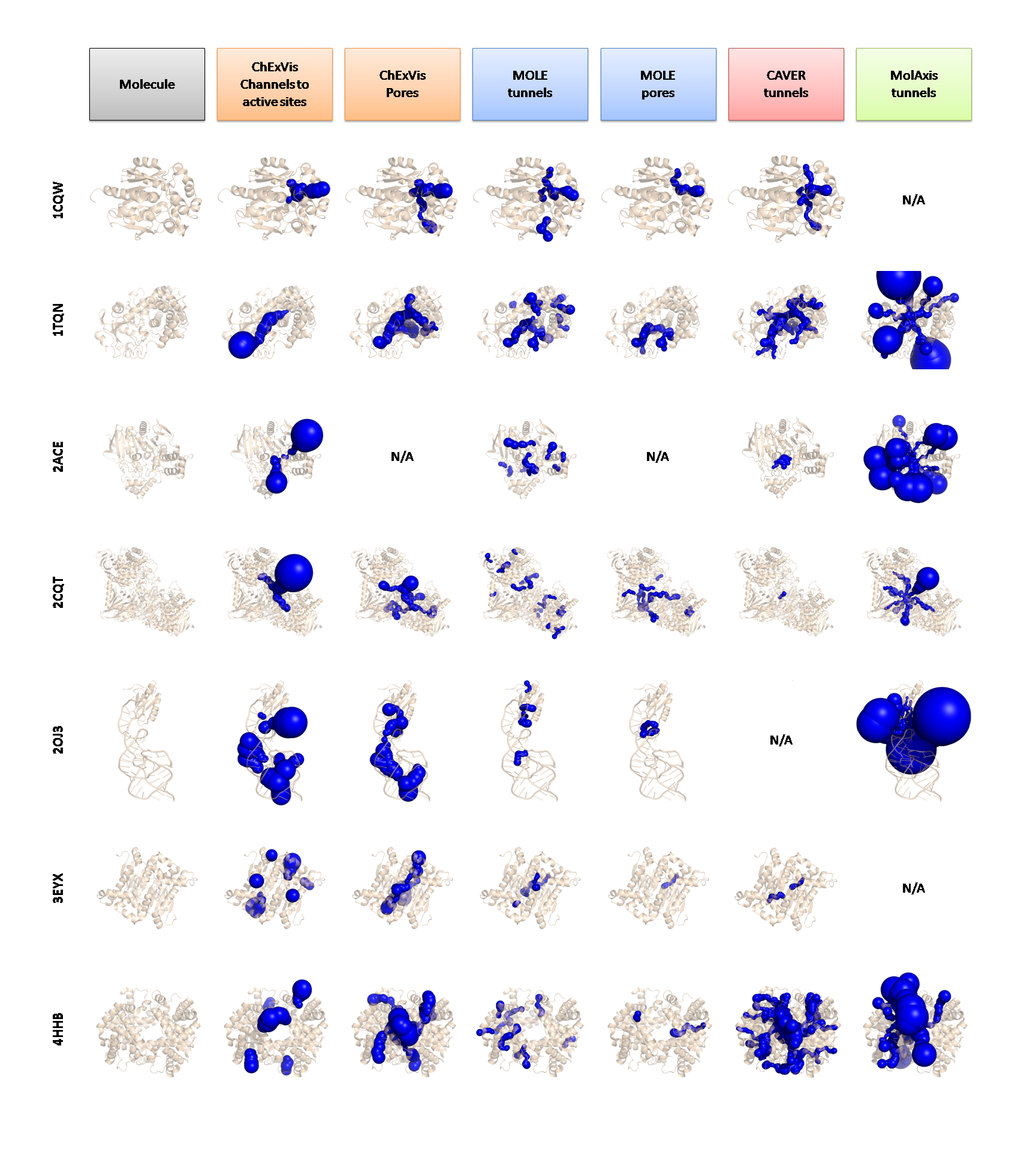

Supplement: Additional file 3 — Comparison of channel extraction tools for enzymes. The results of extraction of channels in a select set of enzymes using different tools viz. ChExVis, Mole, Caver and MolAxis. [file 12859_2015_545_MOESM3_ESM.png]

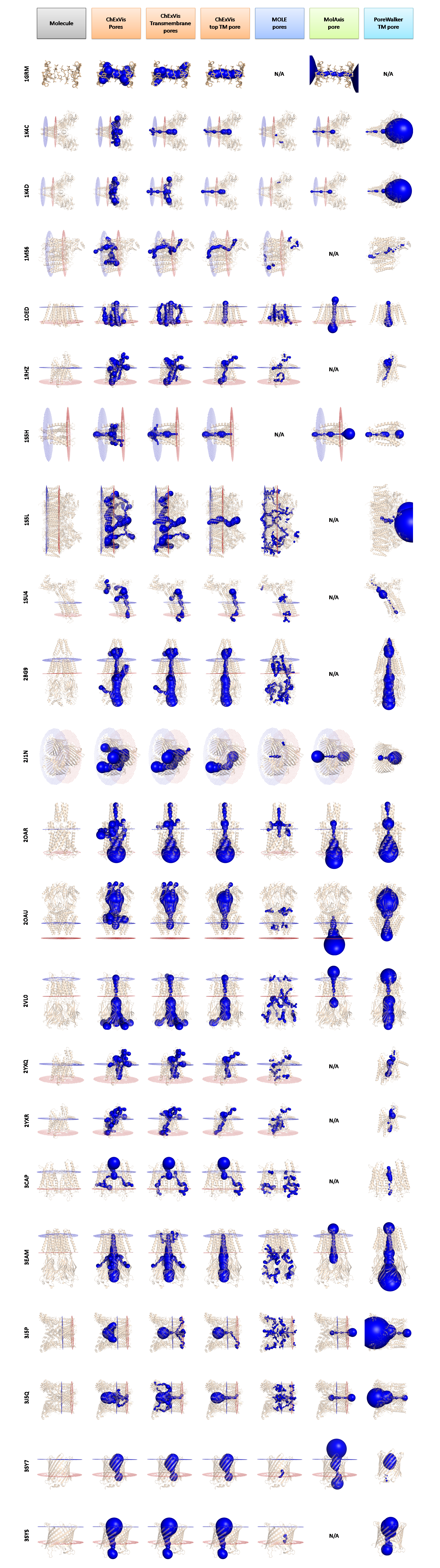

Supplement: Additional file 4 — Comparison of channel extraction tools for transmembrane proteins. The results of extraction of pores in transmembrane proteins using different tools viz. ChExVis, Mole, MolAxis and PoreWalker. [file 12859_2015_545_MOESM4_ESM.png]

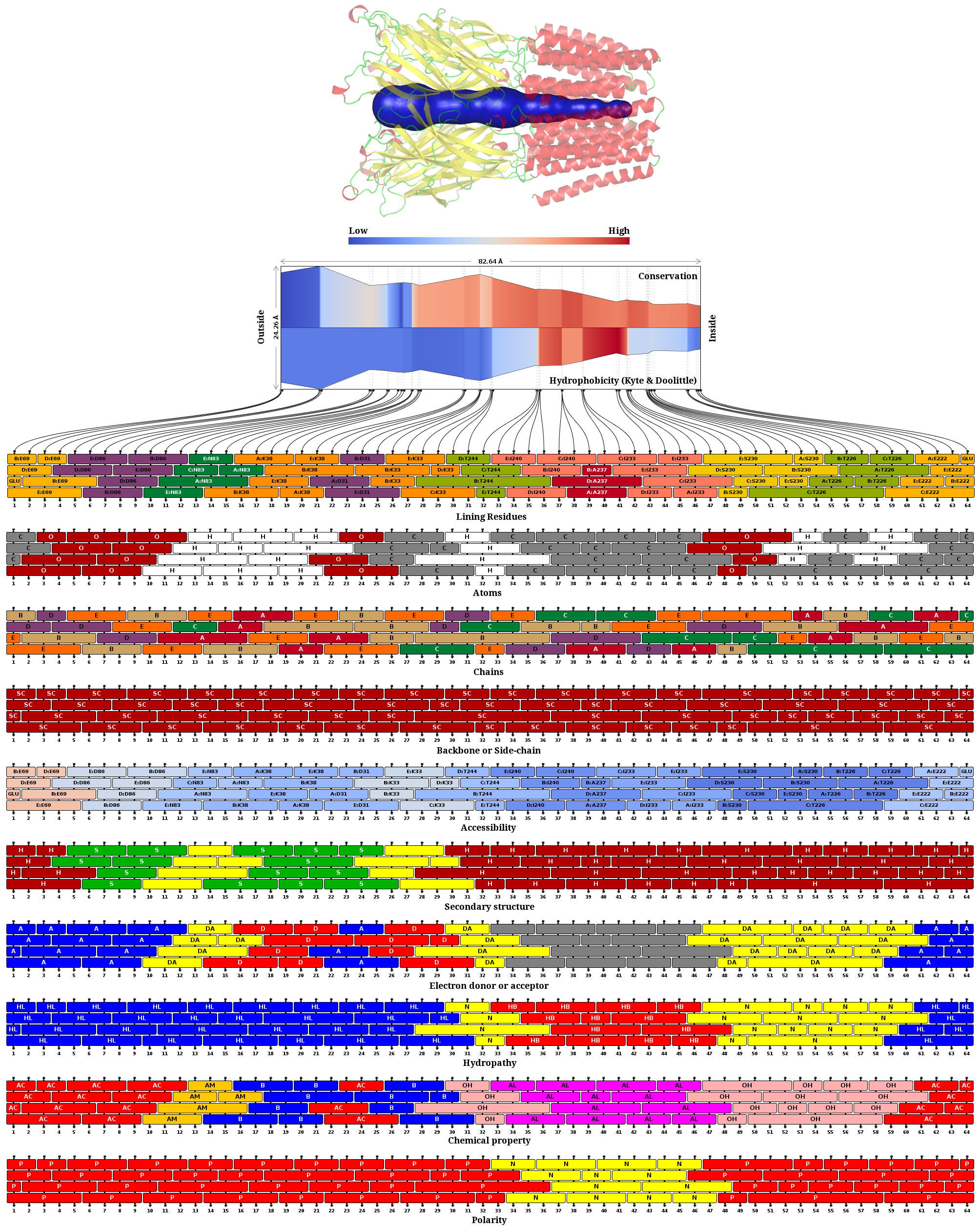

Supplement: Additional file 5 — Properties of pentameric ligand-gated ion channel in the open structure 3EAM. The plots show residues from all five chains that interface with the pore in equivalent positions. The wall of the pore is lined primarily by the side chain atoms of various residues. The view shows that pore interior from positions 32-58 is least accessible while the positions 1-30 are solvent accessible. Channel positions 34-47 lined by residues I240, A237, I233, S230 are hydrophobic and lie on alpha-helices that correspond to the helical wall of the transmembrane spanning regions in the protein. [file 12859_2015_545_MOESM5_ESM.png]

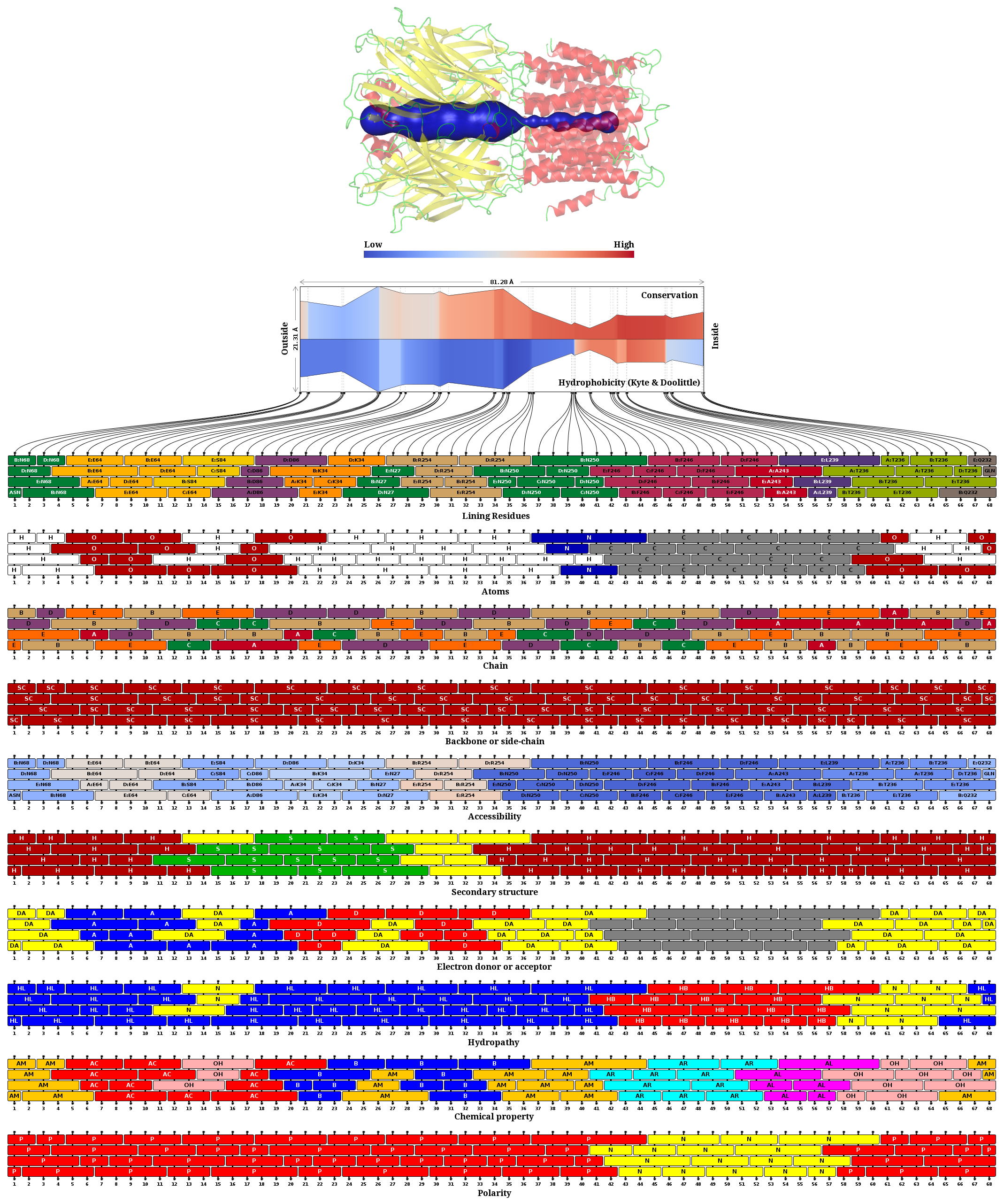

Supplement: Additional file 6 — Properties of pentameric ligand-gated ion channel in the closed structure 2VL0. Compared to channel in open state (3EAM), the channel in 2VL0 is much more constricted at the transmembrane helical region. There is also a drastic reduction in solvent accessibility of this region from 40Å2 to 10Å2. Some changes in chemical properties of the the lining residues are also observable in closed conformation, as N250 and F246 protrude towards the pore around constriction as against I240 and I233 in open state. Also, in these channels the extra-cellular tunnel lined by hydrophilic residues is not very conserved, while the narrow selective region lying in transmembrane region is highly conserved. [file 12859_2015_545_MOESM6_ESM.png]

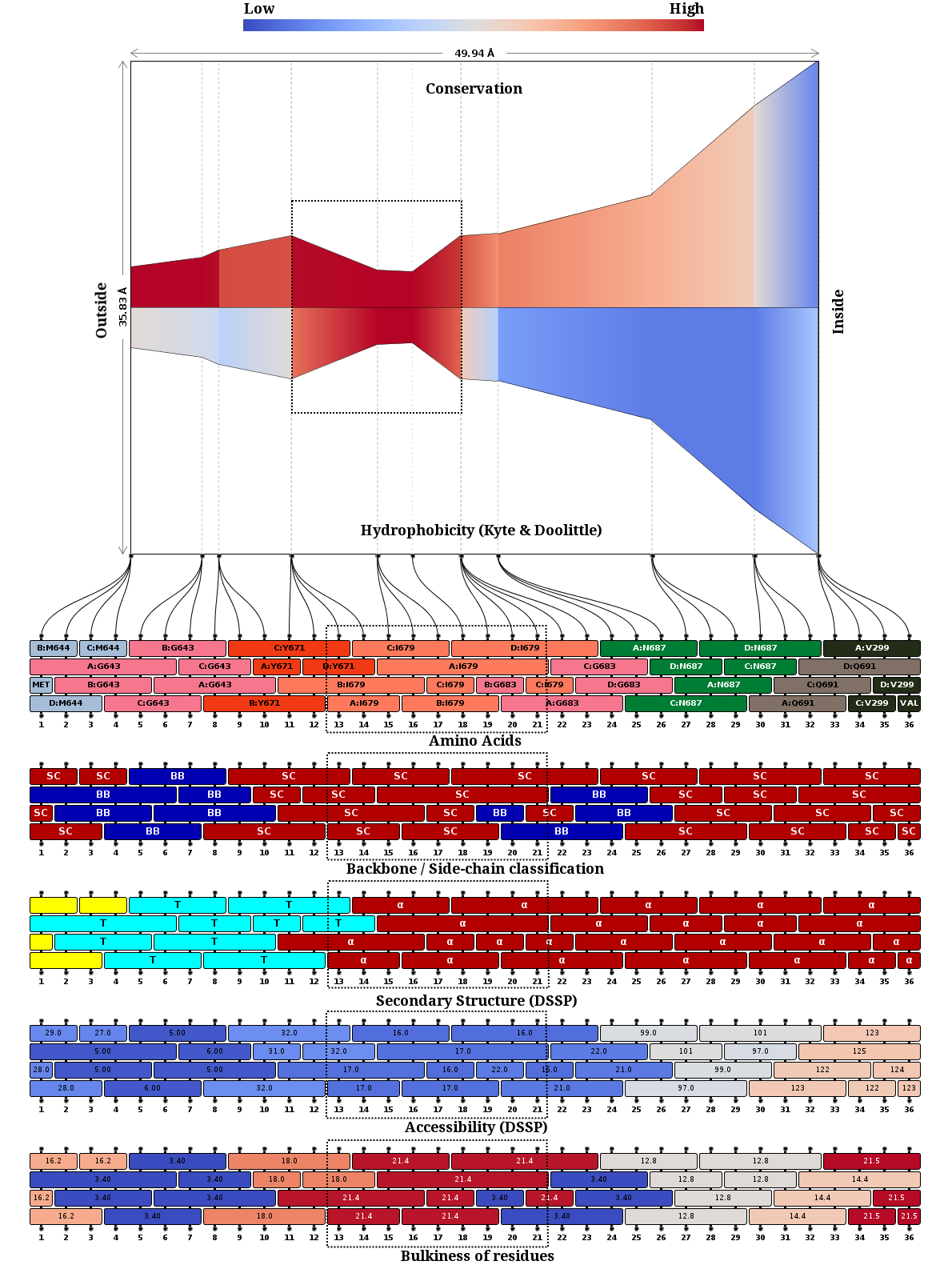

Supplement: Additional file 7 — Properties of transient receptor potential channel in the closed structure 3J5P. In this structure, the radius of the channel is only 2.6Å at the narrowest point. The 2D profile is coloured by conservation and hydrophobicity, red color denoting high values while blue denotes low values. It can be observed the constricted region is highly conserved and hydrophobic. The next five rows of box representations show the properties of amino-acids lining the channel. From this data, it can be concluded that I679 lies at the narrowest point of the channel. Further I679 is a bulky residue with side-chain protruding towards the channel and it has low accessibility. According to third row, most of the residues lining the channel are part of alpha-helices and small helical turns towards the end. [file 12859_2015_545_MOESM7_ESM.png]

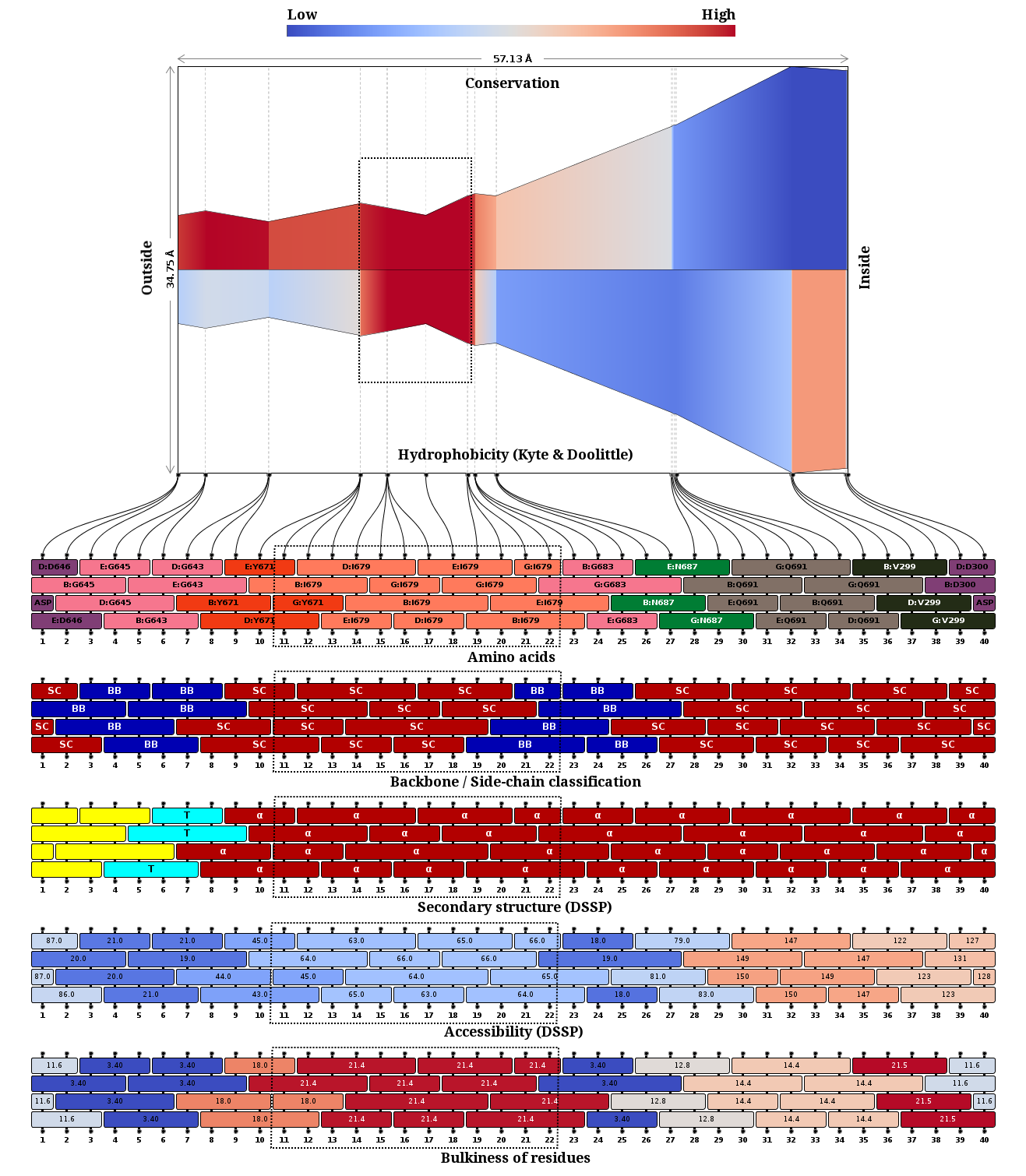

Supplement: Additional file 8 — Properties of transient receptor potential channel in the open structure 3J5Q. In this structure, the radius of the channel is 4.66Å at the narrowest point, which is substantial improvement over closed structure bottleneck radius of 2.6Å. The 2D profile is coloured by conservation and hydrophobicity, revealing that the constricted region is highly conserved and hydrophobic. It can be seen most of residues lining the channel are same as that in 3J5P. Again I679 is an important residue lying at the constriction point. It is important to observe that accessibility of the channel residues in 3J5Q is higher than in closed state i.e. 3J5P. Specially, accessibility of I679 goes up from 16Å2 to 65Å2. [file 12859_2015_545_MOESM8_ESM.png]

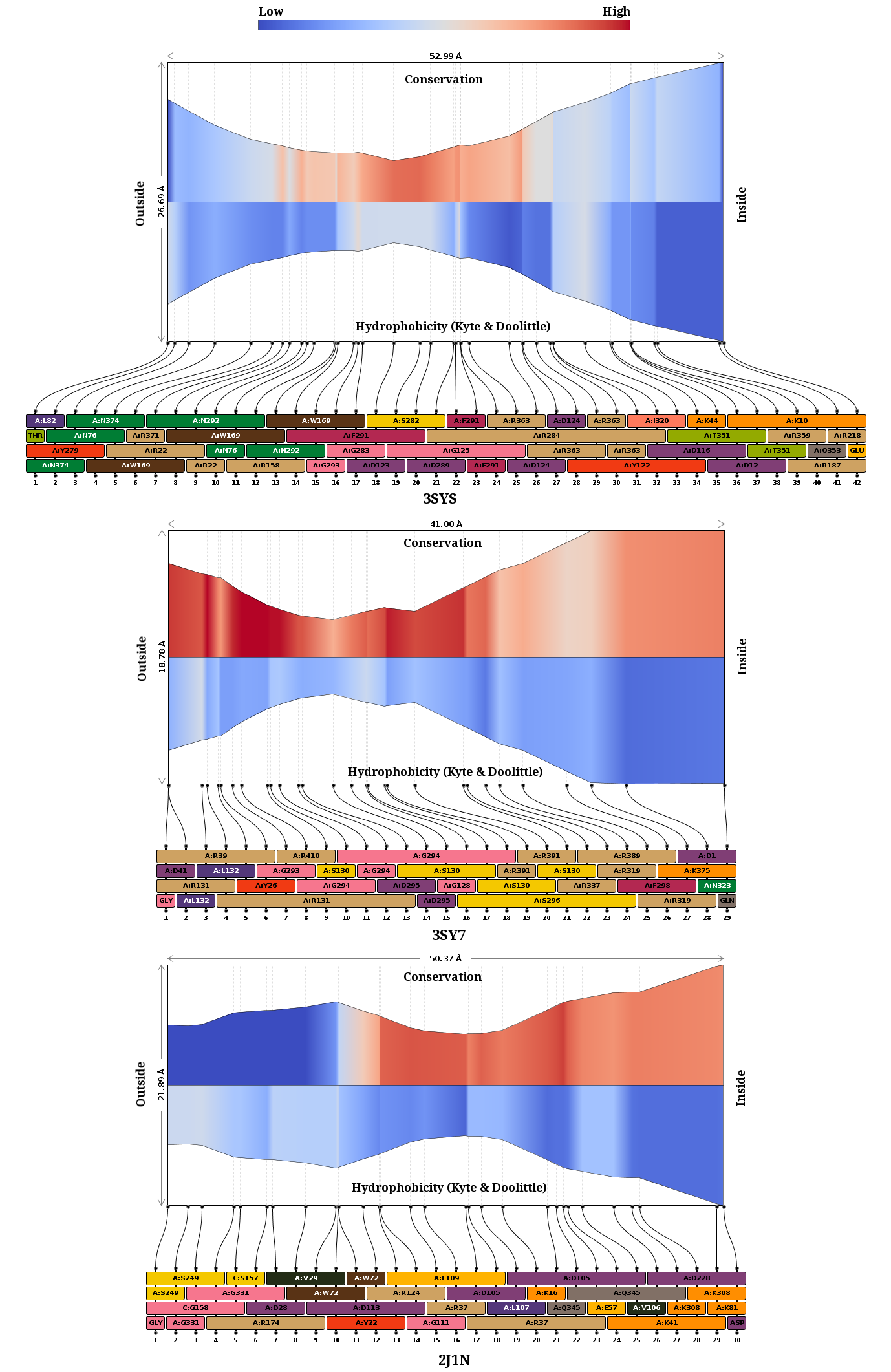

Supplement: Additional file 9 — Comparison of three outer membrane carboxylate channels. The 2D representations of channels from top to bottom correspond to 3SYS, 3SY7 and 2J1N respectively. The split-profiles are coloured by conservation and hydrophobicity of residues, red denoting higher values while blue denotes low values. The amino acids lining these channels are also shown using box representation. A high proportion of basic residue Arginine (coloured as light brown box) in the channel neighbourhood can be clearly observed. [file 12859_2015_545_MOESM9_ESM.png]

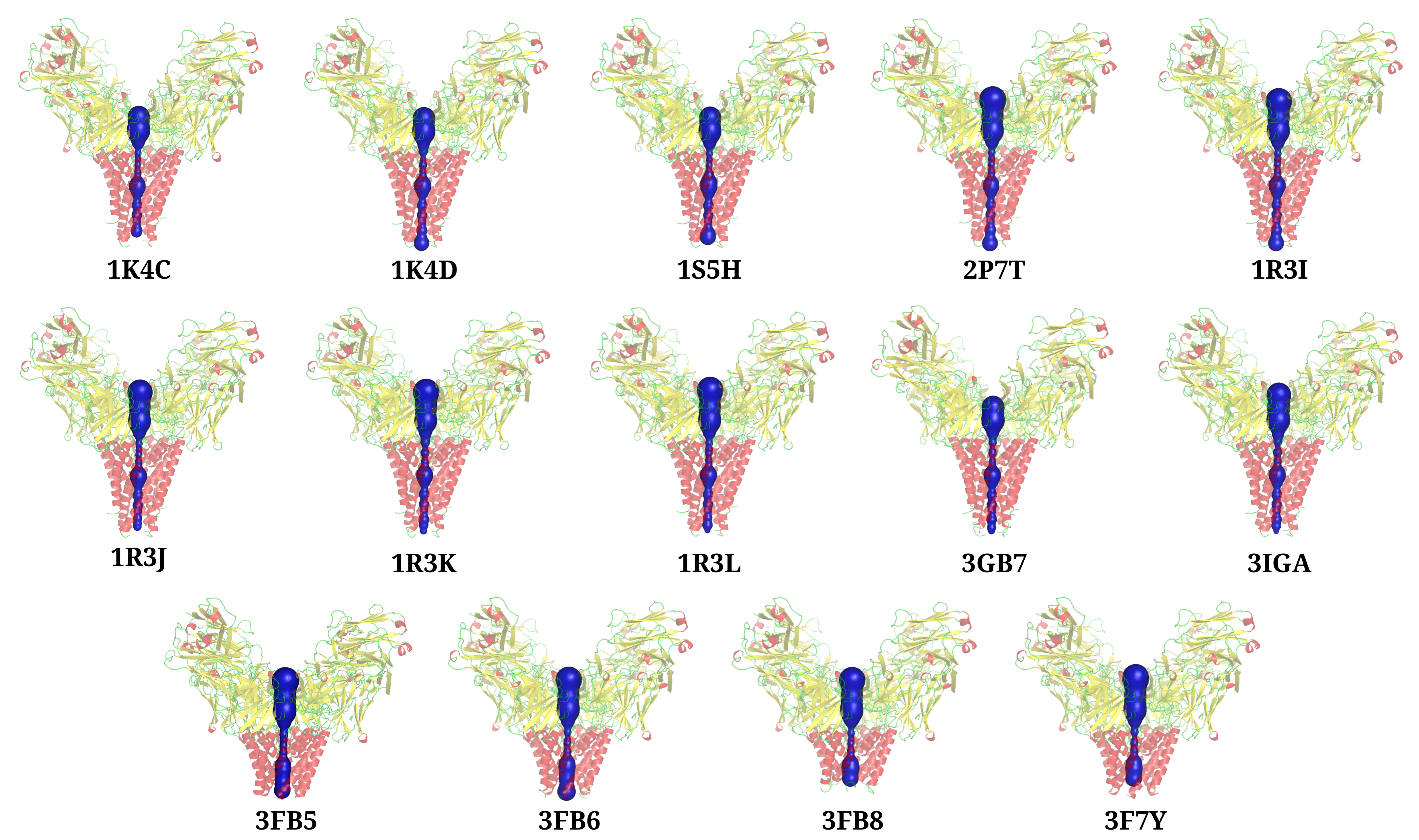

Supplement: Additional file 10 — KcsA channels. KcsA channels extracted in different structures by ChExVis. The PDB ID of the structures are shown below each figure. [file 12859_2015_545_MOESM10_ESM.png]

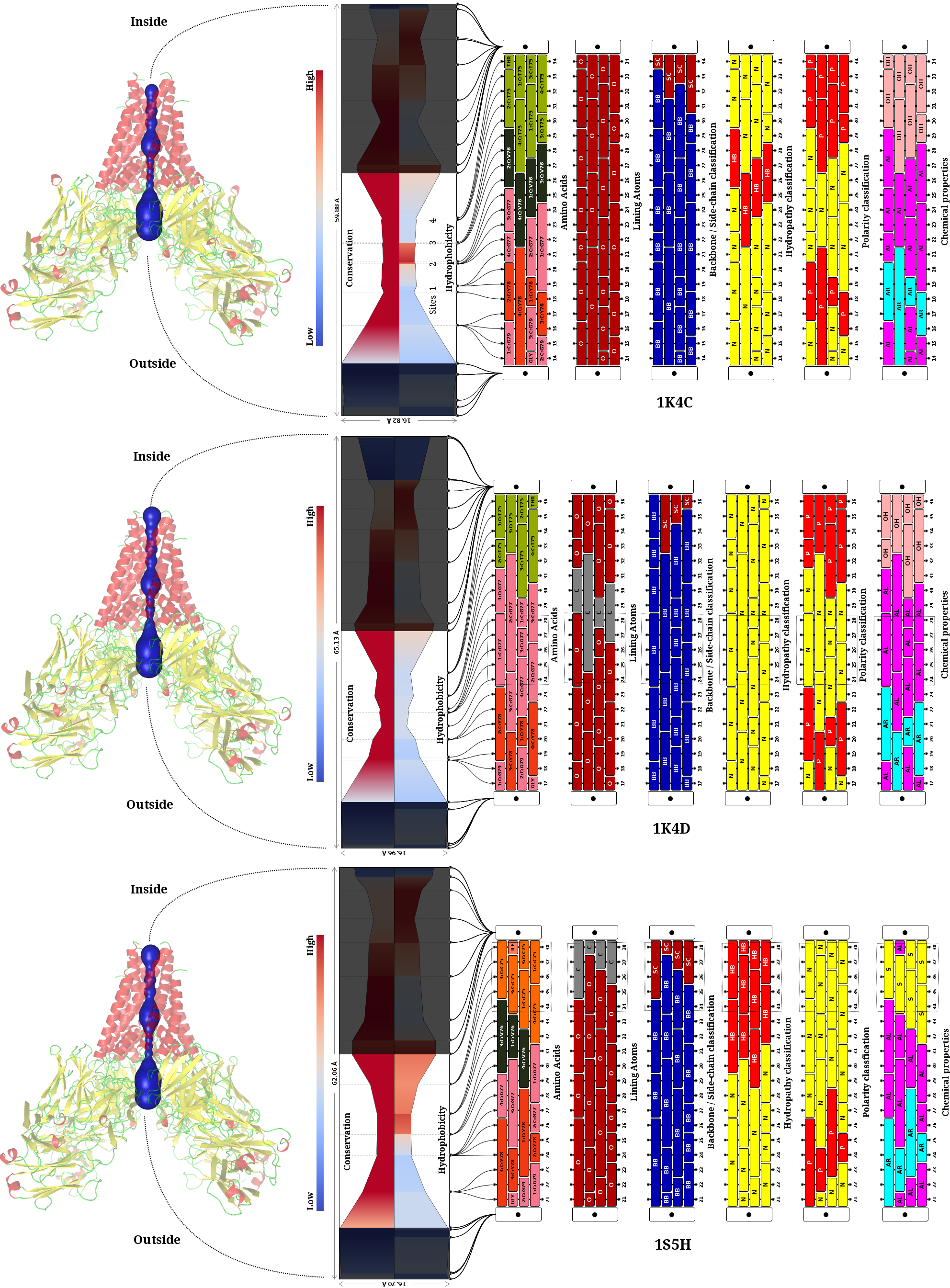

Supplement: Additional file 11 — Comparison of three transmembrane KcsA channels. From top to bottom, properties of channels in 1K4C, 1K4D and mutant 1S5H are shown. The mutation of T75 to C75 is correctly captured by ChExVis as highlighted by a box in 1S5H lining residues. We can clearly observe a few green boxes change to orange in the mutated channel. This mutation also affects the physico-chemical properties of the channel as shown subsequent rows of the above figure. For example, chemical property, hydrophobicity and polarity exhibit clear change. Moreover, in the mutated structure there are fewer Oxygen lining the channel, which are critical for functionality of this transmembrane channel. Also, the channel becomes constricted in lower concentration of K+ ions as captured in 1K4D channel profile. In this configuration, the critical Oxygen atoms are replaced by Carbon atoms as highlighted by a box. [file 12859_2015_545_MOESM11_ESM.png]
